# Caco-2 Cells for Measuring Intestinal Cholesterol Transport - Possibilities and Limitations

**DOI:** 10.1186/s12575-020-00120-w

**Published:** 2020-04-11

**Authors:** Verena Hiebl, Daniel Schachner, Angela Ladurner, Elke H. Heiss, Herbert Stangl, Verena M. Dirsch

**Affiliations:** 1 Department of Pharmacognosy, University of Vienna, Althanstrasse 14, 1090 Vienna, Austria; 2 Institute of Medical Chemistry, Center for Pathobiochemistry and Genetics, Medical University of Vienna, Vienna, Austria

**Keywords:** Caco-2, Cholesterol uptake, Cholesterol efflux, ABCA1, ABCG5/G8, NPC1L1, ABCB1, Transfection

## Abstract

**Background:**

The human Caco-2 cell line is a common in vitro model of the intestinal epithelial barrier. As the intestine is a major interface in cholesterol turnover and represents a non-biliary pathway for cholesterol excretion, Caco-2 cells are also a valuable model for studying cholesterol homeostasis, including cholesterol uptake and efflux. Currently available protocols are, however, either sketchy or not consistent among different laboratories. Our aim was therefore to generate a collection of optimized protocols, considering the different approaches of the different laboratories and to highlight possibilities and limitations of measuring cholesterol transport with this cell line.

**Results:**

We developed comprehensive and quality-controlled protocols for the cultivation of Caco-2 cells on filter inserts in a single tight monolayer. A cholesterol uptake as well as a cholesterol efflux assay is described in detail, including suitable positive controls. We further show that Caco-2 cells can be efficiently transfected for luciferase reporter gene assays in order to determine nuclear receptor activation, main transcriptional regulators of cholesterol transporters (ABCA1, ABCB1, ABCG5/8, NPC1L1). Detection of protein and mRNA levels of cholesterol transporters in cells grown on filter inserts can pose challenges for which we highlight essential steps and alternative approaches for consideration. A protocol for viability assays with cells differentiated on filter inserts is provided for the first time.

**Conclusions:**

The Caco-2 cell line is widely used in the scientific community as model for the intestinal epithelium, although with highly divergent protocols. The herein provided information and protocols can be a common basis for researchers intending to use Caco-2 cells in the context of cellular cholesterol homeostasis.

## Background

The human Caco-2 cell line is a widely used in vitro model of the intestinal epithelial barrier. Caco-2 cells are derived from a colon adenocarcinoma and undergo spontaneous differentiation when kept in post-confluent cultures, developing morphological and biochemical features of polarized small intestinal enterocytes [1–3]. Caco-2 cells are mainly used for assessing the bioavailability of drugs or test compounds [4], but are also suitable for studying lipid and cholesterol homeostasis [5–7], including cholesterol uptake and efflux. The intestine is a major interface in cholesterol uptake and excretion, as shown by its role in transintestinal cholesterol efflux (TICE). For a long time, the hepatobiliary reverse cholesterol transport (RCT) was considered to be the only significant route for cholesterol excretion from the body, until it was discovered that a part of the cholesterol found in feces originates from TICE, representing a non-biliary pathway for eliminating cholesterol via the small intestine (reviewed in [8]). Liver X receptors (LXRs), which form an obligate and permissive heterodimer with retinoid X receptors (RXRs), are master regulators of cholesterol homeostasis and were shown to increase fecal sterol excretion upon activation. LXR activation in the intestine upregulates the transporter heterodimer ATP-binding cassette (ABC) G5/G8, leading to increased cholesterol efflux into the intestinal lumen, and decreases the expression of Niemann-Pick C1-like protein 1 (NPC1L1), thereby limiting cholesterol uptake. LXR activation in the intestine further upregulates ABCA1, leading to increased cholesterol efflux to apolipoprotein A1, which results in the formation of high density lipoprotein (HDL), highlighting the importance of the intestine in cholesterol turnover (reviewed in [8]).

Many publications exist, which use the Caco-2 cell model. The available descriptions of the used methodology, however, are either undetailed or not consistent [1, 4, 9–12], as described in more detail in the results part. This hampers the kick-off or reproduction of experiments with this cell line. Our objective was therefore to generate a detailed and optimized protocol collection, considering the different approaches of the different laboratories and evaluating and modifying the conditions to achieve a reliable and valid outcome.

## Results

### Maintenance of Caco-2 Cells and Cultivation on Filter Inserts with Spontaneous Differentiation

First, we validated protocols for the maintenance of Caco-2 cells (purchased from DSMZ, Braunschweig, Germany) and for cultivation conditions that lead to the formation of a tight monolayer when cells are seeded onto filter inserts. Cultivation of Caco-2 cells on filter inserts leads to an improved functional and morphological differentiation, making it the method of choice for most assays [2]. Moreover, it enables access to both the apical and the basolateral side of the cells and allows the usage of different media for these two sides, closer resembling the physiological situation. Different protocols are already available for cultivating Caco-2 cells on filter inserts [1, 2, 4, 9, 10, 13–17]. They, however, differ in used seeding densities, membrane materials and duration of differentiation. We used the available protocols as a basis [1, 2, 4, 9, 10, 13–17] and tested several parameters that differed between these publications (polycarbonate and PET membrane materials without/with coating (collagen and gelatine), different seeding densities, different periods of differentiation). We monitored the formation of a cell monolayer under these different test conditions and found that the cell seeding density and the period of differentiation are especially crucial for the outcome. When we seeded cells at a higher density (especially above 0.2 × 10^6^ per cm^2^), we observed confluence earlier and detachment of cells after 16 days in cultivation, as judged by transepithelial electrical resistance (TEER) measurement. Gelatine or collagen coating did not change this result. Therefore, we reduced cell seeding density, which allows differentiation for 21 days without detachment. We found that cultivation periods over 21 days increase the likelihood of a detachment of the cells, as judged by a rapid decrease in TEER. Cultivation for 19–21 days was finally chosen since a study by Briske-Anderson et al. suggested that 18–21 days of cultivation leads to optimal enzymatic properties and uniformity in cell density [13].

Regarding cell culture material, we found that both polycarbonate and PET inserts are suitable for cultivation. We decided to use PET inserts for all other studies, since they are available as both translucent and transparent membranes, whereas polycarbonate membranes are available in translucent form only.

Several quality controls, described in the following paragraphs, were used to evaluate the integrity of the monolayer, finally yielding valid protocols, detailed in the methods section (Protocol #1, Protocol #2, Supplementary Protocol #1 (Additional file 1) and Supplementary Protocol #2 (Additional file 1)).

**Fig. 1:**
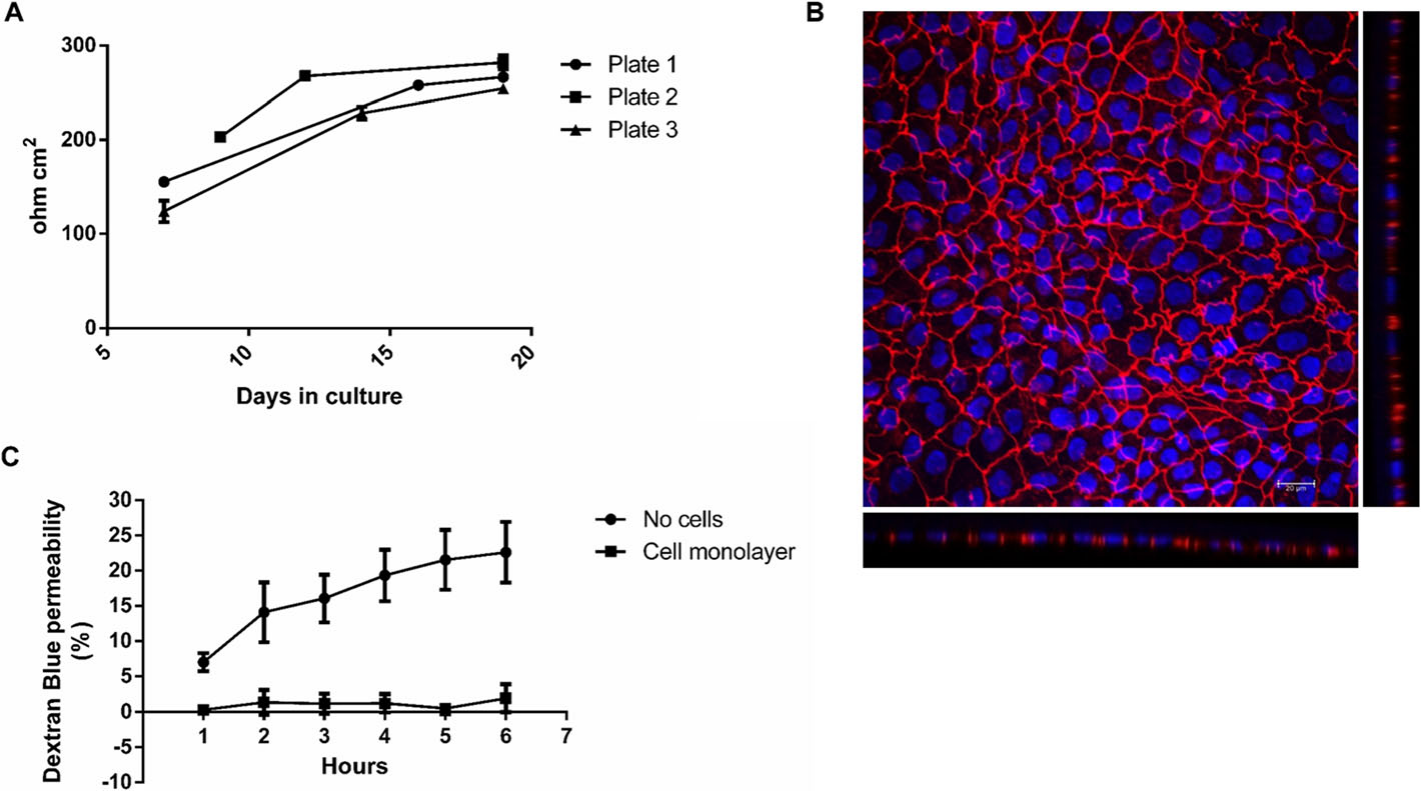
**a–c** Quality control steps for the cultivation of Caco-2 cells on filter inserts. Increase in TEER over the cultivation period (**a**). Caco-2 cells were seeded onto filter inserts applying Protocol #2 and TEER was measured as detailed in Supplementary Protocol #3 (Additional file 1). The data points represent mean ± SD from three inserts from the respective plate. Representative image of a Caco-2 cell monolayer grown on a filter insert for 21 days (**b**). Depicted is a maximum projection of the top view and the optical cross sections obtained by CLSM. Cell nuclei are shown in blue (DAPI) and the tight junction protein ZO-1 in red (Alexa Fluor 594 conjugated anti ZO-1 antibody). Staining was carried out as described in Protocol #6. Dextran Blue permeability of a filter insert without cells and a filter insert with a fully differentiated cell monolayer (**c**). Filter inserts were incubated with Dextran Blue as described in Supplementary Protocol #4 (Additional file 1) over a period of 6 h and sampling every hour. The data points represent mean ± SD from three to four independent experiments

First of all, TEER, detailed in Supplementary Protocol #3 (Additional file 1), was measured at least once a week in order to monitor and guarantee the integrity of the monolayer under the used conditions and before starting any experiments. A typical increase in TEER values is shown in Fig. 1a. For experiments, we used inserts only with a TEER value of ≥200 Ω cm^2^ at 37 °C, which is in accordance with an already published study [4] and can easily be achieved with the used cultivation conditions.

In addition, confocal laser scanning microscopy (CLSM) at the end of the differentiation period verified formation of a plain and intact monolayer under the used cultivation conditions. Staining for CLSM was performed as described in Protocol #6, using a fluorescently labelled antibody against the tight junction protein zonula occludens (ZO)-1 (Alexa Fluor 594 conjugated anti-ZO-1 antibody, 1 μg/ml (1:500)) and DAPI (4′,6-diamidine-2′-phenylindole dihydrochloride) to stain cell nuclei. A representative image of a Caco-2 cell monolayer grown on a filter insert can be seen in Fig. 1b in both top view and cross section, which shows the presence of a single, tight cell layer under the used conditions.

Another very common and useful parameter to ensure the integrity of the formed monolayer is the paracellular permeability for the markers Dextran Blue or [^14^C]-mannitol. The paracellular permeability for these compounds was tested as described in Supplementary Protocol #4 (Additional file 1). The paracellular transit of Dextran Blue was observed every hour over a total period of 6 h, and the results are shown in Fig. 1c. The permeability for [^14^C]-mannitol was assessed over a period of 2 h. Typical apparent permeability values for [^14^C]-mannitol in our laboratory are 1.30 ± 0.77 × 10^− 6^ cm s^− 1^.

### Transfection of Caco-2 Cells and Luciferase Reporter Gene Assay

**Fig. 2:**
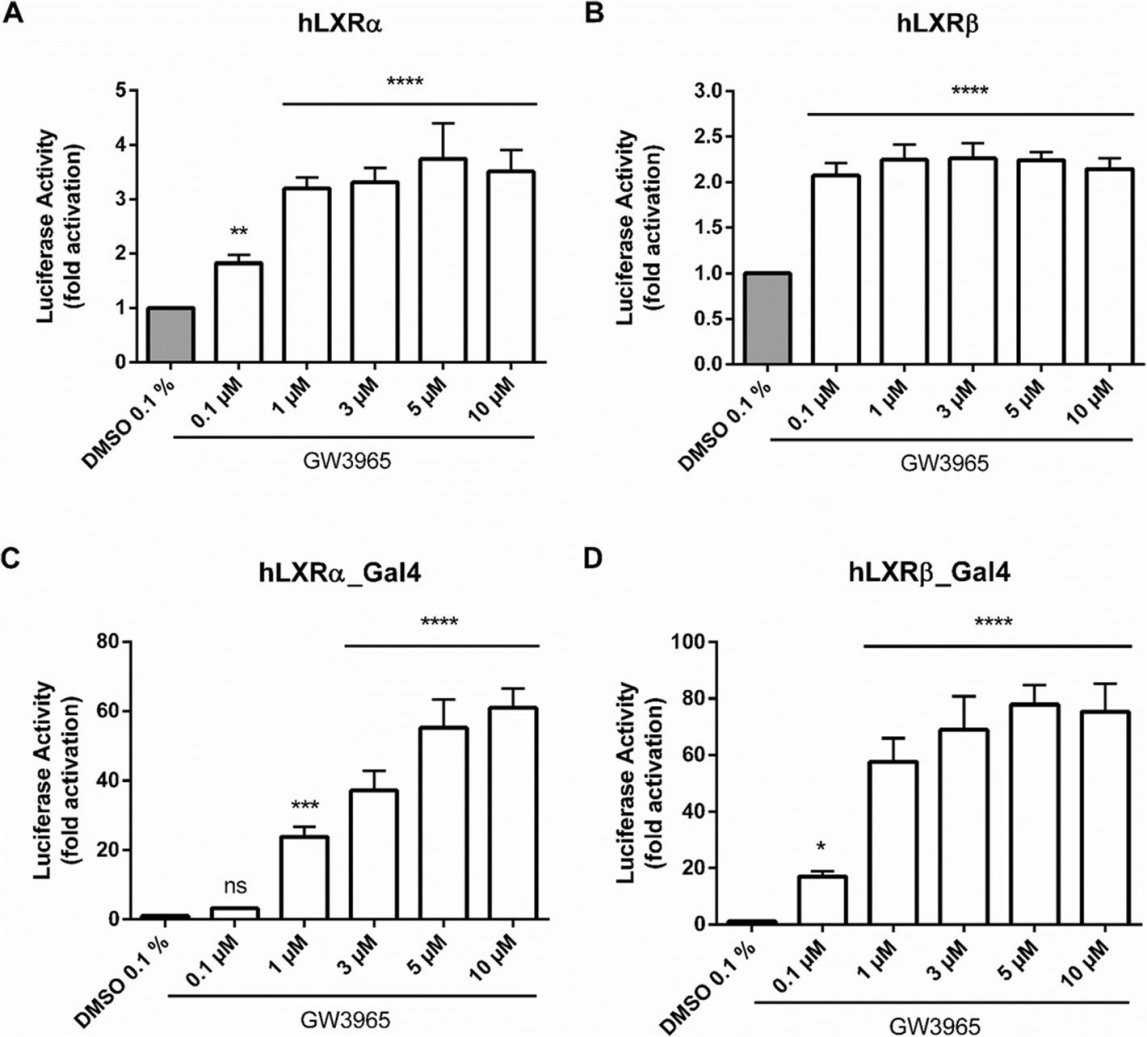
**a–d** Luciferase Reporter Gene Assays with Caco-2 cells, applying Protocol #3. Caco-2 cells were transiently co-transfected with a human full-length LXRα (**a**) or LXRβ (**b**) expression plasmid, the firefly luciferase reporter plasmid ABCA1_Luc and an EGFP expression plasmid, in a ratio of 1:3:1, using Lipofectamine™ LTX. In the case of the mammalian one-hybrid assays, cells were transiently transfected with an expression plasmid encoding a chimeric protein consisting of the yeast Gal4-DBD fused in frame to the hLXRα-LBD (**c**) or to the hLXRβ-LBD (**d**), a reporter plasmid containing specific upstream activating sequences (UAS)_Luc, and the EGFP expression plasmid, in a ratio of 5:3:1. Cells were then treated with GW3965 (LXR agonist, 0.1–10 μM) or the vehicle control (DMSO 0.1%). The luciferase-derived luminescence was normalized to the EGFP-derived fluorescence, and set in relation to the solvent vehicle. Bar graphs represent mean ± SD from three independent experiments each performed in quadruplicate. ns not significant, *P < 0.05, **P < 0.01, ***P < 0.001, ****P < 0.0001 versus solvent vehicle (determined by one-way ANOVA with Dunnett post-test)

Several nuclear receptors have crucial implications in the regulation of cholesterol homeostasis (reviewed in [18, 19]). Determining the activation of distinct nuclear receptors is therefore of high interest in the field of cholesterol and atherosclerosis research. A wide-spread assay used to investigate nuclear receptor activation is the luciferase reporter gene assay, which is usually carried out in HEK293 cells [20], as they are easy to transfect. Nonetheless, (organ) specific cellular features like the expression of co-regulatory proteins that affect gene expression may be unique in intestinal Caco-2 cells (reviewed in [21]). We, therefore, decided to transfect Caco-2 cells and to establish a protocol for a luciferase reporter gene assay in this cell line (Protocol #3). Since differentiated cells are far more resistant to transfection [22], undifferentiated cells were used for transfection and the subsequent luciferase reporter gene assay. Several groups already successfully transfected Caco-2 cells, however, the used transfection reagents differed between these groups. We found publications using FuGENE® 6, Lipofectamine™ 2000, calcium phosphate coprecipitation, PEI (polyethylenimine) and other methods, including different nanoparticles for improved gene delivery [23–27]. We, therefore, decided to test several transfection reagents. From all tested transfection reagents, i.e. FuGENE® HD, the calcium phosphate precipitation method [20, 28] and LPEI (linear polyethylenimine), only Lipofectamine™ LTX led to an acceptable transient transfection efficiency as judged by the green fluorescence provided by the internal control EGFP. We then adapted the protocol of the manufacturer of Lipofectamine™ LTX regarding the number of cells seeded, the amount of Lipofectamine™ LTX used per well and the ratios of the expression, reporter and internal control plasmids transfected. With the cell seeding density recommended in our protocol, approximately 50% confluence is reached when transfection is started, which we found appropriate to achieve a high transfection efficiency and to avoid excessive cell death. In general, high confluence is associated with a reduced transfection efficiency, but a low seeding number can result in increased cell death during transfection [29]. We co-transfected the cells with a human full-length LXRα or LXRβ expression plasmid, the firefly luciferase reporter plasmid ABCA1_Luc and an EGFP expression plasmid, which served as an internal control, in a ratio of 1:3:1. For the mammalian one-hybrid assay, cells were transiently transfected with an expression plasmid encoding a chimeric protein consisting of the yeast Gal4-DBD fused in frame to the hLXRα/β-LBD, a reporter plasmid containing specific upstream activating sequences (UAS)_Luc, and the EGFP expression plasmid, which again served as internal control, in a ratio of 5:3:1. Fig. 2a-d shows the results of cells treated with increasing concentrations of the LXR agonist GW3965. Luciferase activity was significantly induced in a dose-dependent manner, except for hLXRβ, which showed a plateau from 1 μM GW3965 upwards. Altogether, the results show that Caco-2 cells can be efficiently transfected for luciferase reporter gene assays using Lipofectamine™ LTX.

### Establishing Functional Assays with Caco-2 Cells for Cholesterol Uptake and Cholesterol Efflux

Since the intestine is an important interface for cholesterol uptake and excretion, we set up functional assays for studying e.g. the influence of test compounds on cholesterol uptake and efflux. Cholesterol uptake and efflux assays were already carried out by different groups, but the described methodology differs in regard to several assay conditions, i.e. differentiation of Caco-2 cells on plates with/without inserts, incubation with cholesterol packaged in micelles or not, composition of the micelles, incubation time and use/no use of acceptors like apoA1 [30–34]. Therefore, we also tested and adjusted these conditions for the cholesterol uptake and efflux assay.

**Fig. 3:**
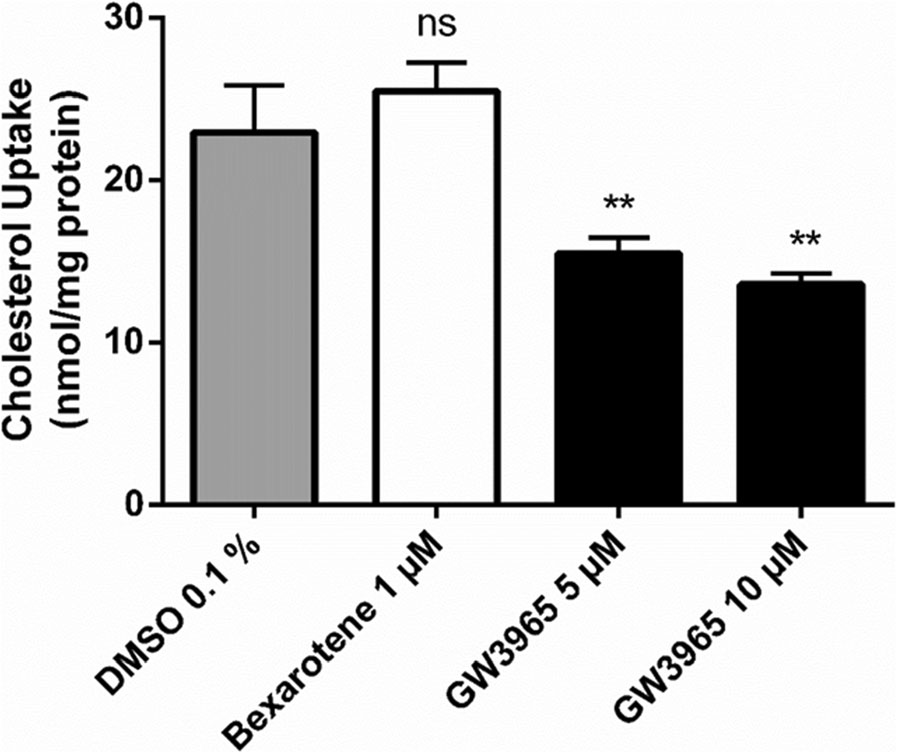
Cholesterol Uptake Assay with Caco-2 cells, applying Protocol #4. Caco-2 cells cultivated on filter inserts were treated for 48 h with bexarotene (RXR agonist, 1 μM), GW3965 (LXR agonist, 5 and 10 μM) or the vehicle control (DMSO 0.1%). Cells were then incubated with micelles containing [^3^H]-cholesterol and fresh treatment for 2 h on the apical side, while being incubated with 1% human plasma on the basolateral side. Cholesterol uptake was then determined by liquid scintillation counting. Protein concentration was measured and the results expressed as nmol cholesterol/mg protein before being normalized for interexperimental variance. Bar graphs represent mean ± SD from three independent experiments. ns not significant, **P < 0.01 versus solvent vehicle (determined by paired t-test or one-way ANOVA with Dunnett post-test)

For the cholesterol uptake assay (Protocol #4), cells were cultivated and fully differentiated on filter inserts. Cells were treated for 48 h with either the LXR agonist GW3965 (5 or 10 μM) or the RXR agonist bexarotene (1 μM). After the treatment period, cells were exposed to micelles mimicking postprandial conditions [35, 36] and fresh treatment on the apical side, while being incubated with 1% human plasma on the basolateral side for 2 h. Hereafter, the uptake of radioactively labelled cholesterol was determined. As expected for a LXR agonist, GW3965 significantly reduced cholesterol uptake at both concentrations used (Fig. 3). In contrast, treatment with the RXR agonist bexarotene did not result in a decrease of cholesterol uptake.

**Fig. 4:**
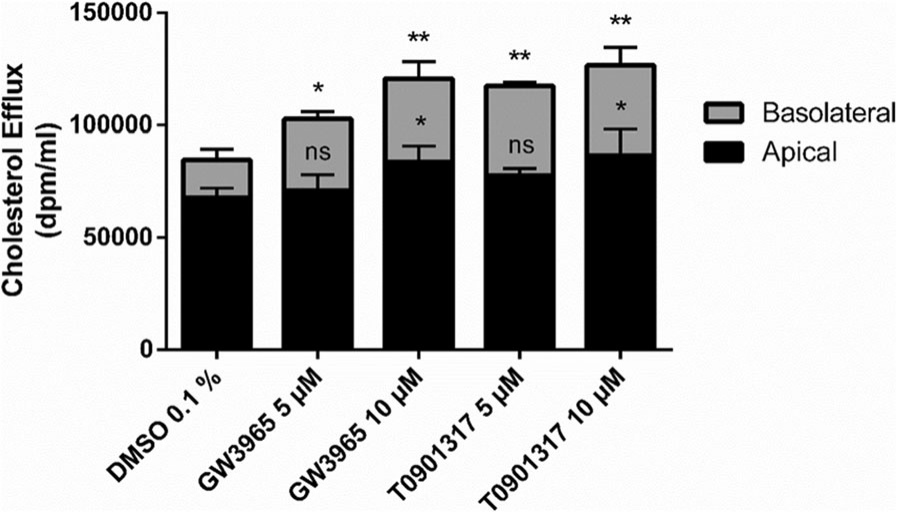
Cholesterol Efflux Assay with Caco-2 cells, using Protocol #5. Caco-2 cells cultivated on filter inserts were loaded with cholesterol by incubation with micelles containing [^3^H]-cholesterol for 24 h. Hereafter, cells were treated for 48 h with GW3965 (LXR agonist, 5 and 10 μM), T0901317 (LXR agonist, 5 and 10 μM) or the vehicle control (DMSO 0.1%). Finally, cells were incubated with cholesterol-free micelles on the apical side, while being incubated with 1% human plasma on the basolateral side. Effluxed (apical and basolateral supernatant) cholesterol was then measured by liquid scintillation counting. The results in dpm were normalized to 1 ml volume, before being normalized for interexperimental variance. Bar graphs represent mean ± SD from three to four independent experiments. ns not significant, *P < 0.05, **P < 0.01 versus solvent vehicle (determined by one-way ANOVA with Dunnett post-test)

Like in the cholesterol uptake assay, Caco-2 cells grown on filter inserts were used for the cholesterol efflux assay (Protocol #5). Cells were exposed to micelles with radioactively labelled cholesterol for 24 h (cholesterol loading) and then incubated with different LXR agonists (GW3965 and T0901317 both at 5 and 10 μM) for 48 h. Hereafter, cells were incubated with cholesterol-free micelles on the apical, and 1% human plasma on the basolateral side for 24 h. Effluxed (apical and basolateral supernatant) cholesterol was then measured by liquid scintillation counting. As shown in Fig. 4, both GW3965 and T0901317 led to a concentration-dependent increase in cholesterol efflux. Treatment with these LXR agonists increased the amount of cholesterol effluxed to both the apical and the basolateral side.

### Detection of Protein and mRNA Levels of the Cholesterol Transporters ABCA1, ABCG5, NPC1L1 and ABCB1

In intestinal enterocytes, there are several transporters that are responsible for the uptake and efflux of cholesterol, namely ABCA1, the heterodimer ABCG5/G8, NPC1L1 and ABCB1 (reviewed in [8, 37]). The effect of a compound or drug on the mRNA and/or protein expression of these transporters is therefore of relevance for cholesterol transport studies. The cholesterol transporters ABCA1, ABCG5/G8, NPC1L1 and the rather unspecific transporter ABCB1 (better known as P-glycoprotein) are either up- or downregulated, directly or indirectly, by the activation of nuclear receptors (reviewed in [8, 38]).

We cultivated Caco-2 cells on filter inserts and treated cells with either GW3965 or T0901317 (LXR agonists, 10 μM, upregulating ABCA1 and ABCG5/G8, downregulating NPC1L1 (reviewed in [8])), or bexarotene (RXR agonist, 5 μM, co-regulating LXR:RXR or CAR:RXR heterodimers (reviewed in [39])). Due to co-regulation of LXR:RXR heterodimers, we expected to detect an upregulation of ABCA1 and ABCG5/G8, as well as a downregulation of NPC1L1 also with bexarotene. Since ABCB1 expression is regulated via permissive CAR:RXR heterodimers, among others (reviewed in [38]), we also expected to see an upregulation of this protein with bexarotene treatment.

**Fig. 5:**
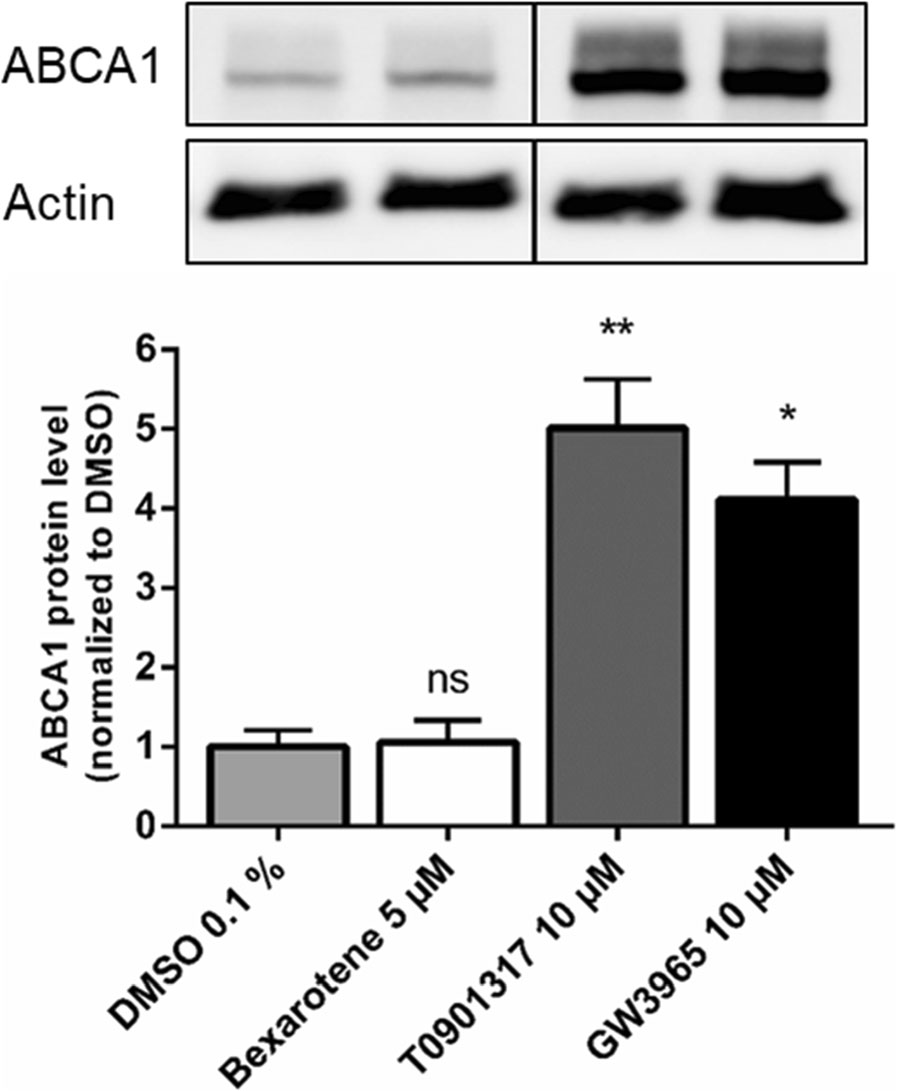
Western Blotting for ABCA1, as detailed in Supplementary Protocol #5 (Additional file 1). Caco-2 cells cultivated on filter inserts were treated for 48 h with bexarotene (RXR agonist, 5 μM), T0901317 (LXR agonist, 10 μM), GW3965 (LXR agonist, 10 μM) or the vehicle control (DMSO 0.1%). Protein expression was then determined by western blot analysis. Results were normalized for interexperimental variance and then set in relation to the vehicle control. Bar graphs represent mean ± SD from three independent experiments. A representative blot is shown - the bands stem from the same membrane and the vertical line indicates excised irrelevant bands. ns not significant, *P < 0.05, **P < 0.01 versus solvent vehicle (determined by paired t-test)

Time course experiments (4 h to 72 h) with subsequent western blot analysis (Supplementary Protocol #5 (Additional file 1)) showed an induction of ABCA1 by about 4-fold and 5-fold after 48 h of treatment with GW3965 and T0901317, respectively, but no induction with bexarotene (Fig. 5). We concluded that the use of the NP40 lysis buffer and a treatment time of 48 h are seminal conditions to investigate the protein level of ABCA1 with western blot analysis. The finding that bexarotene does not increase the protein expression of this bona fide LXR target gene in Caco-2 cells is in contrast to findings e.g. in glial cells, where bexarotene significantly induces ABCA1 expression [40]. For ABCG5, ABCG8, NPC1L1 and ABCB1 data were inconsistent (data not shown) throughout all conditions tested (detailed discussion in the Discussion section). The use of a different lysis buffer (RIPA instead of NP40) did not improve the outcome. For this reason, we switched to protein detection via CLSM, applying Protocol #6 for staining. Several protocols for staining and CLSM with Caco-2 cells are already available [17, 41–43]. However, fixation of cells, the blocking solution used as wells as the permeabilization differ between these publications. Moreover, to the best of our knowledge, only two publications were detecting the NPC1L1 protein in Caco-2 cells via CLSM [42, 43], whereas no publications exist regarding ABCG5 and ABCB1. Therefore, we provide an adjusted and reliable protocol for the use of CLSM for detecting cholesterol transporters in Caco-2 cells.

**Fig. 6:**
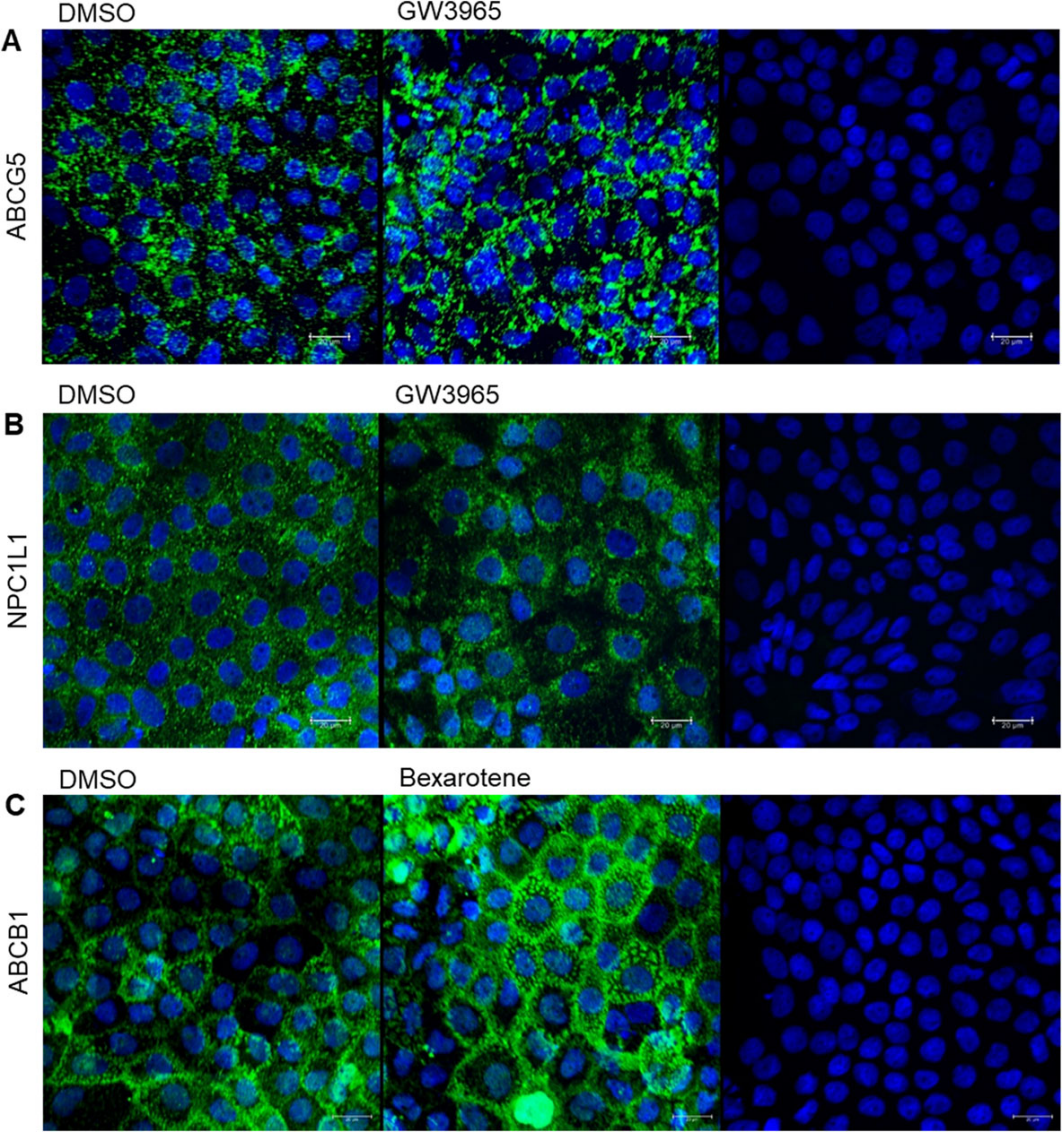
**a–c** Confocal laser scanning microscopy for the detection of ABCG5, NPC1L1 and ABCB1. Staining was carried out using Protocol #6. Cell nuclei are shown in blue (DAPI) and ABCG5, NPC1L1 or ABCB1 in green (fluorescently labelled antibody). Representative images of ABCG5 (**a**), treated with the vehicle control (DMSO 0.1%, left image) or GW3965 (LXR agonist, 10 μM, middle image). Representative images of NPC1L1 (**b**), treated with the vehicle control (DMSO 0.1%, left image) or GW3965 (LXR agonist, 10 μM, middle image). Representative images of ABCB1 (**c**), treated with the vehicle control (DMSO 0.1%, left image) or bexarotene (RXR agonist, 5 μM, middle image). The image on the right (**a–c**) is the respective control only stained with DAPI and the secondary antibody, showing the unspecific binding of the secondary antibody. Shown are maximum projections of the top views

By CLSM, we were able to detect an upregulation of ABCG5 and ABCB1, and a downregulation of NPC1L1, when treating cells for 48 h with the respective positive controls, as shown in Fig. 6a-c. Thus, we conclude, at least for the Caco-2 strain and the conditions used and tested in our laboratory, that western blot analysis seems less suitable for the detection of cholesterol transporters, whereas CLSM appears to be a good alternative.

For the analysis of mRNA expression, we cultivated Caco-2 cells on filter inserts and treated them with either GW3965 (LXR agonist, 10 μM), or bexarotene (RXR agonist, 5 μM). Treatment was carried out over different intervals, namely 2 h, 4 h, 6 h, 8 h, 16 h, 24 h and 30 h, in order to detect the time point of maximal upregulation or downregulation. After treatment, cells were subjected to RNA extraction and qRT-PCR as described in Supplementary Protocol #6 (Additional file 1). For the extraction of RNA, a commercial kit (peqGOLD total RNA kit) was used first, which, however, yielded RNA with very poor quality and meagre amounts of RNA (less than 50 ng/μl). Therefore, we then used TRIzol for RNA extraction, resulting in acceptable RNA quality and sufficient amounts of RNA (above 100 ng/μl) for subsequent qRT-PCR.

**Fig. 7:**
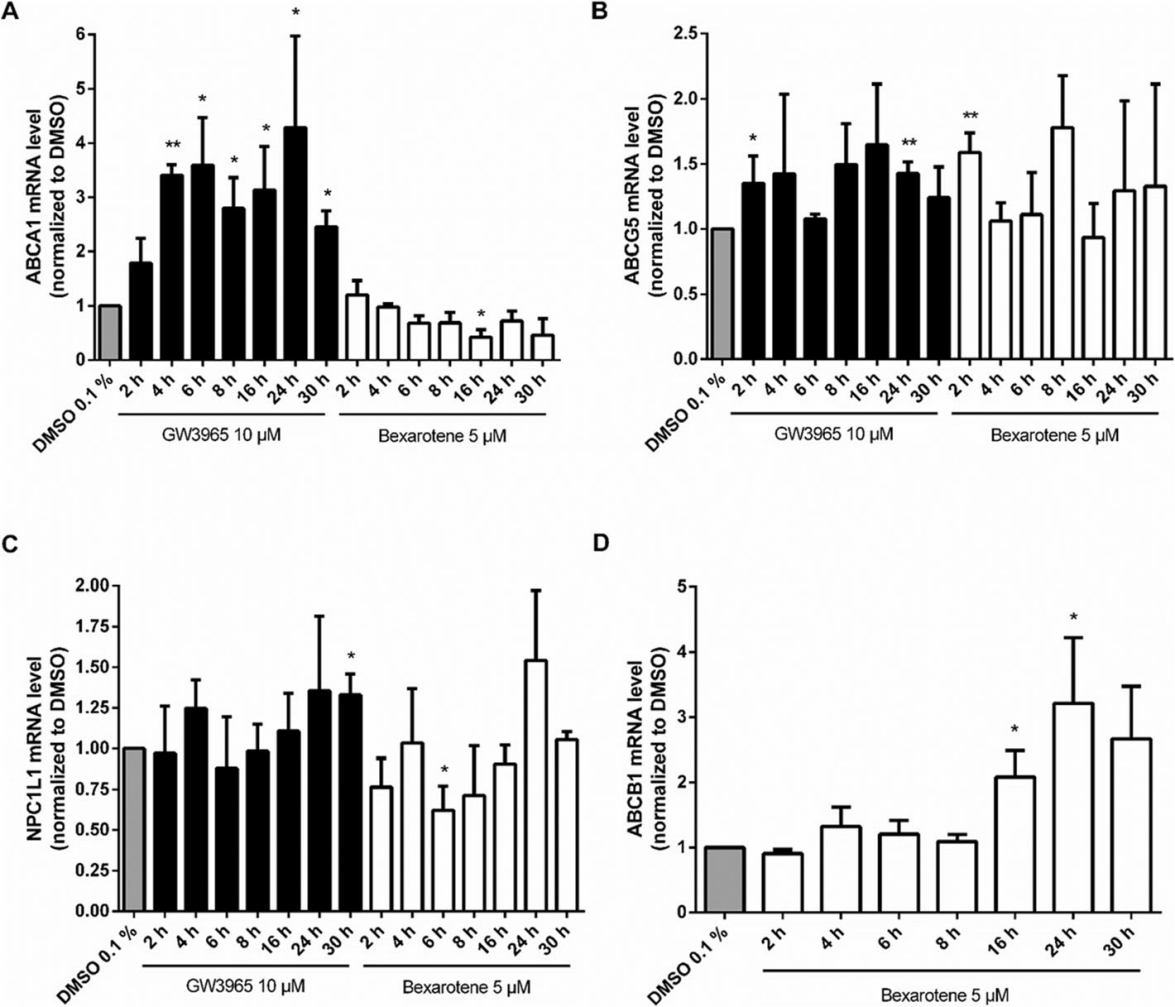
**a–d** qRT-PCR with Caco-2 cells for ABCA1, ABCG5, NPC1L1 and ABCB1. Caco-2 cells cultivated on filter inserts were treated for the indicated time points with bexarotene (RXR agonist, 5 μM), GW3965 (LXR agonist, 10 μM) or the vehicle control (DMSO 0.1%). Extraction of RNA and quantification of mRNA expression by qRT-PCR was carried out as described in Supplementary Protocol #6 (Additional file 1). Results were expressed as the ratio of expression of the respective gene to that of GAPDH and were then normalized to the vehicle control of the respective time point (vehicle control not indicated for each time point). Bar graphs represent mean ± SD from three to four independent experiments. *P < 0.05, **P < 0.01 versus solvent vehicle (determined by paired t-test)

GW3965 significantly increased ABCA1 mRNA level as early as 4 h after treatment and a significant upregulation was maintained throughout the test period (Fig. 7a). Notably, bexarotene did not induce ABCA1 mRNA expression at any of the time points. Instead there was a trend towards inhibition that reached significance after 16 h treatment. This contrasts findings in other cell types as e.g. brain-like endothelial cells [44].

ABCG5 mRNA level were significantly increased by treatment with GW3965 for 2 h and for 24 h, as well as by treatment with bexarotene for 2 h (Fig. 7b). All other time points showed a decline in ABCG5 mRNA expression or highly variable mRNA levels, hampering statistical significance. In general, induction of ABCG5 was low, with about 1.4-fold after 24 h treatment with GW3965 compared to DMSO.

The LXR agonist GW3965 did not downregulate NPC1L1 at any of the time points (2–30 h) (Fig. 7c), which is in contrast to already published work [45] that reports a significant decrease after 24 h of treatment in Caco-2/TC7 cells (see Discussion). Interestingly, bexarotene caused a downregulation of NPC1L1 on the mRNA level. Treatment for 2 h, 8 h and 16 h showed a tendency of reduced NPC1L1 mRNA expression, and 6 h of treatment with bexarotene significantly reduced NPC1L1 expression.

Bexarotene induced ABCB1 mRNA expression significantly after 16 h and 24 h of treatment (Fig. 7d). Maximal upregulation of ABCB1 by about 3-fold compared to the solvent vehicle was observed after 24 h of treatment with bexarotene.

### Viability Assays with Caco-2 Cells Cultivated on Filter Inserts

Viability Assays are a standard procedure to assure that a certain compound does not affect cell viability. Since most of the assays described in our protocol collection are performed with cells on filter inserts, we set up a protocol for a resazurin conversion assay and a subsequent crystal violet staining with cells cultivated on inserts (Protocol #7).

**Fig. 8:**
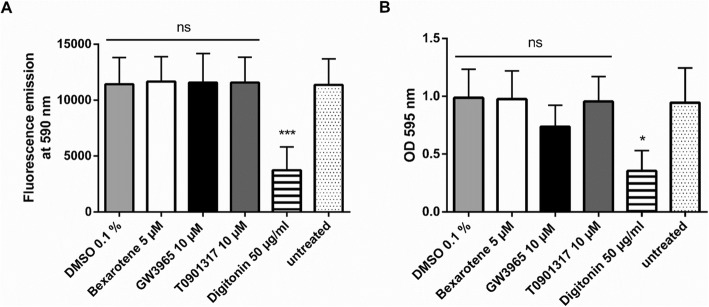
**a** and **b** Resazurin Conversion Assay and subsequent Crystal Violet staining with Caco-2 cells grown on filter inserts. Protocol #7 describes the steps of the assays. Caco-2 cells were either left untreated (negative control) or were treated for 48 h with GW3965 (LXR agonist, 10 μM), T0901317 (LXR agonist, 10 μM), bexarotene (RXR agonist, 5 μM), digitonin (positive control, 50 μg/ml) or the vehicle control (DMSO 0.1%). Cells were then incubated with a dilution of resazurin in serum-free DMEM (10 μg/ml) for 2 h. Hereafter, fluorescence emission was measured in the apical medium (**a**). The inserts were then incubated with crystal violet solution for 10 min. After washing steps and drying overnight, crystal violet was solubilized using sodium citrate:ethanol and the OD was measured at 595 nm (**b**). Bar graphs represent mean ± SD from three independent experiments. ns not significant, *P < 0.05, ***P < 0.001 versus untreated (determined by paired t-test)

Fully differentiated Caco-2 cells were either left untreated in serum-free DMEM or were treated for 48 h with the highest concentrations of the compounds used in previous experiments, i.e. GW3965 (10 μM), T0901317 (10 μM), bexarotene (5 μM) and the vehicle control (DMSO 0.1%). As a positive control for cytotoxicity, we used the natural product digitonin at 50 μg/ml. After the treatment period, cells were washed with PBS and then incubated for 2 h with a resazurin dilution (10 μg/ml in serum-free DMEM). Aliquots of the apical medium were then used for the measurement of fluorescence (excitation 535 nm, emission 590 nm) in a plate reader. When compared to the untreated cells, digitonin significantly reduced fluorescence, i.e. cell viability, in the apical supernatant (Fig. 8a). The other compounds did not affect fluorescence in the apical medium.

After the detection of fluorescence, inserts were incubated with a crystal violet solution for 10 min, followed by extensive washing steps with deionized water and drying overnight. Crystal violet was then solubilized with the help of sodium citrate:ethanol and OD was measured at 595 nm. Digitonin significantly reduced the absorbance, while none of the other compounds had a significant effect (Fig. 8b). These results indicate that the used concentrations of the compounds are not toxic to the cells.

## Discussion

The Caco-2 cell line is a suitable in vitro model to study cholesterol transport and expression of cholesterol transporters. Cells are easy to maintain and differentiate spontaneously when kept in culture after confluence [1–3]. It is known that cultivation of Caco-2 cells on filter inserts leads to an improved morphological and functional differentiation, which suggests the use of cells differentiated on filter inserts [2]. This also gives access to and allows the use of different media for the apical and the basolateral side of the cells, closer resembling the physiological situation. When cultivated on filter inserts, several quality control steps are, however, needed in order to assure the integrity of the monolayer and to avoid the formation of multilayers. CLSM is a suitable tool to check the presence of a single layer of cells and to observe tight junction formation. Moreover, TEER measurement and the investigation of the paracellular permeability for compounds like Dextran Blue and [^14^C]-mannitol are standard techniques used to evaluate the quality of the formed monolayer. It has to be mentioned, however, that TEER and apparent permeability values vary highly with passage number and the clone or strain used [13, 46–49]. For experiments in our laboratory, we only used monolayers on inserts with a TEER value of ≥200 Ω cm^2^ at 37 °C. In our Dextran Blue permeability assay, we used 2.5% human plasma as acceptor in the basolateral compartment, since human plasma is also used as an acceptor in both the cholesterol uptake and the cholesterol efflux assay. Since publications exist using up to 2.5% human plasma [50], we decided to test this concentration in the Dextran Blue permeability assay, although this is slightly higher compared to the concentration we use. With this we sought to exclude an influence of human plasma on the integrity of the monolayer.

To determine nuclear receptor activation, we established an adjusted and valid protocol for a luciferase reporter gene assay in Caco-2 cells. For this, several different transfection reagents, i.e. FuGENE® HD, the calcium phosphate precipitation method [20, 28], LPEI and Lipofectamine™ LTX were tested. In our hands, only Lipofectamine™ LTX led to a suitable transfection efficiency, as judged by the green fluorescence provided by the internal control EGFP. Although LXR functions as heterodimer with RXR (reviewed in [18]), we transfected expression plasmids for LXR only. Endogenous RXR protein expression, however, was verified by western blot analysis (data not shown). In addition, experiments in Fig. 2 prove that overexpression of LXR is sufficient to detect activation by LXR agonists.

Two protocols for functional assays were established that show the suitability of this cell line to study both cholesterol uptake and cholesterol efflux. LXR agonists are appropriate positive controls in both assays, leading to decreased cholesterol uptake and to increased cholesterol efflux.

Although we were able to detect ABCA1 protein level via western blot analysis, we were unable to obtain convincing data for the other transporters, even though a treatment time course (4 to 72 h) was performed (data not shown) and two different lysis buffers were used (NP40 versus RIPA). Blots showed blurred bands and/or multiple bands in most of the experiments, making a semi-quantitative analysis very difficult. This is likely due to the fact that the cholesterol transporters we wanted to detect are glycoproteins, existing in non-glycosylated and different glycosylated isoforms during the process of maturation [51–54].

In the case of ABCG5/G8, LXR response elements were found in two evolutionary conserved regions distal of the intergenic promoter, regulating ABCG5/G8 expression [55]. It was shown that another transcription factor, liver receptor homolog-1 (LRH-1), binds to the intergenic promoter [56]. However, treatment of Caco-2 cells with an LRH-1 agonist (DLPC (dilauroyl phosphatidlycholine)) [57] did not consistently alter the protein expression level of ABCG5 either. Also the use of antibodies targeting both partners in this heterodimer, i.e. ABCG5 and ABCG8, did not overcome the obstacles. Since ABCG5/G8 are glycosylated proteins, samples were treated with the endoglycosidase PNGase F to cleave sugar residues, but this also did not improve results. Studies showing western blot analysis of ABCG5/G8 originate mainly from in vivo experiments in mice. The few studies using Caco-2 cells did in most cases not include positive controls, i.e. LXR agonists. To the best of our knowledge, we identified only two studies that included positive controls in Caco-2 cells [31, 58], but did not state where the Caco-2 cells originate from, which makes it difficult to directly compare their results to ours. Noteworthy, both studies did not cultivate Caco-2 cells on filter inserts as we did, which may explain the different outcome.

Since activation of PPARδ leads to a downregulation of NPC1L1 in intestinal cells [59, 60], we incubated cells with the commercially available PPARδ agonist GW0742 and checked the protein level of NPC1L1, which, however, did not lead to convincing results. As for ABCG5/G8, available comparative data stem mainly from experiments in mice, and the few Caco-2 studies in most cases lack suitable positive controls. For NPC1L1, we also found two studies that included positive controls in Caco-2 cells [58, 61], but again did not mention the Caco-2 cell source and cultivated cells without filter inserts.

Concerning ABCB1, we used valproic acid as an alternative positive control, since valproic acid was shown to induce ABCB1 expression via CAR [62]. However, also valproic acid did not lead to consistent and convincing results.

This experience led us to switch to CLSM, as shown in Fig. 6a-c. For CLSM, no enzymatic reaction is required to cleave sugar residues, since all isoforms can be detected at once without separation via gel electrophoresis. Moreover, a semi-quantitative analysis of the fluorescence detected can also be performed with specific software tools.

Since a commercial kit (peqGOLD total RNA kit) yielded RNA with very poor quality, which is probably due to the material of the filter inserts, we provide here a protocol for RNA extraction with TRIzol. Fig. 7 a and d show that detecting mRNA levels of ABCA1 and ABCB1 can easily be done using this protocol. More challenging is the identification of the best time point for detecting an up- and downregulation of ABCG5 and NPC1L1, respectively. Concerning ABCG5, the induction achieved by treatment with GW3965 was generally low. Although a general trend of an upregulation was visible, most of the time points did not reach statistical significance. Similar to the protein level analyses, studies addressing ABCG5/G8 mRNA expression in Caco-2 cells are scarce and again most of these studies did not include appropriate positive controls. One study that included positive controls [31] did, however, not mention the origin of Caco-2 cells and did not cultivate them on filter inserts. Notably, it was shown that intestinal cells, also Caco-2 cells, are subjected to a circadian rhythm, as they express several important clock genes [63–67]. Moreover, a circadian regulation of the expression of ABCG5/G8 has already been shown in the liver [68], which is very likely to hold true also for the intestine. The high variability of ABCG5/G8 expression that we observed could be explained by this circadian regulation.

Contrary to already published work [45], we were not able to detect a downregulation of NPC1L1 mRNA at any of the time points during treatment with the LXR agonist GW3965 (Fig. 7c). One possible reason for the discrepancy may be the use of different Caco-2 clones. As mentioned above, the expression of co-regulatory proteins varies among different cell types, which can have an impact on gene expression regulation by nuclear receptors. Thus, we cannot exclude that the expression of co-activators and co-repressors is different between the Caco-2 strain we used and the Caco-2/TC7 clone used by Duval et al. [45]. Nonetheless, treatment with bexarotene for 6 h significantly reduced NPC1L1 mRNA levels. To the best of our knowledge, however, the exact mechanisms that regulate NPC1L1 expression including the role of LXR are still not clear. From five studies addressing NPC1L1 mRNA expression in Caco-2 cells including positive controls [45, 58, 61, 69, 70], only the study by Duval et al. [45] differentiated Caco-2 cells on filter inserts. Notably, circadian regulation of NPC1L1 expression has been reported [71, 72], which could possibly explain the high degree of variability that we observed.

Despite the encountered obstacles we still consider it more appropriate to cultivate Caco-2 cells on filter inserts also for the detection of mRNA and protein levels of cholesterol transporters, since this resembles the situation of the two functional assays and more importantly, the physiological situation.

Interestingly, bexarotene did not reduce cholesterol uptake and is therefore inadequate as positive control in this assay. Neither did bexarotene upregulate ABCA1 protein nor mRNA levels. The latter was even decreased. This is in contrast to findings in other cell types, such as glial cells, brain-like endothelial cells and macrophages [40, 44, 73]. In a study investigating the potential of bexarotene in atherosclerosis, the mRNA levels of both NPC1L1 and ABCA1 were found to be reduced in the duodenum and jejunum of bexarotene-treated mice, supporting our findings. As also discussed in this study, bexarotene seems to act in a tissue-specific and gene-specific manner [73].

Finally, we also set up viability assays with Caco-2 cells cultivated on filter inserts. To the best of our knowledge, no protocol exists for a resazurin conversion assay and a subsequent crystal violet staining with Caco-2 cells differentiated on filter inserts. Both resazurin conversion assay and crystal violet staining are feasible with cells cultivated on filter inserts. However, the washing steps after crystal violet staining have to be very gentle, in order not to detach cells that are still viable and adherent. Since most of the assays in our protocol collection are performed with cells cultivated on filter inserts, we think it is more appropriate to also conduct the viability assays with cells on filter inserts.

Despite the use of filter inserts and different media for the apical and the basolateral side, mimicking the physiological situation, the Caco-2 cell model has several shortcomings. Enterocytes are only one of the four main cell types found in the intestine [74, 75]. No mucus-producing goblet cells or hormone-secreting enteroendocrine cells are present in the Caco-2 in vitro model, meaning that it does not entirely reflect cholesterol handling in vivo. Several groups have suggested to co-culture Caco-2 cells with mucus-producing HT29 cells to improve this cell model. Publications analyzing the advantages and disadvantages of this co-culture have already been published [76–80]. Whenever interpreting results obtained with the Caco-2 cell model, these limitations should be kept in mind.

## Conclusions

The intestine plays a central role in cholesterol homeostasis, highlighting the importance of the Caco-2 cell model for cholesterol research. We present here a collection of optimized protocols for the use of the Caco-2 cell line and show the suitability of this cell line for evaluating intestinal cholesterol uptake and excretion. To analyze protein levels of several cholesterol transporters, western blot analysis appeared largely unsuitable (except for ABCA1). CLSM, however, is a suitable alternative. Care has to be taken in regard to choosing an appropriate positive control and time point for measuring mRNA level of the cholesterol transporters NPC1L1 and ABCG5. Functional assays for cholesterol transport appear more meaningful compared to the evaluation of protein and mRNA level of cholesterol transporters, as they enable an overall impression of how cholesterol is handled under compound treatment or a certain condition investigated.

## Methods

### Materials

Caco-2 cells were obtained from DSMZ (Braunschweig, Germany). All chemicals were purchased from Carl Roth (Karlsruhe, Germany) or Sigma-Aldrich (Vienna, Austria), unless otherwise stated. PET translucent and transparent filter inserts (both 0.4 μM pore size) for 12-well plates, 12-well companion plates and 75 cm^2^ flasks were obtained from Sarstedt (Nuembrecht, Germany). EMEM, DMEM, L-glutamine, penicillin/streptomycin mixture (potassium penicillin/streptomycin sulfate) and 100x NEAA were purchased from Lonza Group Ltd. (Basel, Switzerland). FBS and trypsin were obtained from Gibco via Invitrogen (Lofer, Austria). Human plasma was obtained from healthy, young volunteers. OptiMEM was acquired from Thermo Fisher Scientific (Vienna, Austria). D-[2-^14^C]-mannitol, [1,2-^3^H(N)]-cholesterol, liquid scintillation cocktail (Ultima Gold™) and liquid scintillation vials (MidiVials) were obtained from Perkin Elmer (Traiskirchen, Austria). SYBR® Green I Master and cOmplete™ protease inhibitor cocktail were purchased from Roche Diagnostics (Vienna, Austria). FuGENE® HD transfection reagent and reporter lysis 5x buffer were acquired from Promega (Mannheim, Germany). Luciferin was purchased from Synchem (Felsburg, Germany). TRIzol, RNase-free glycogen, Phasemaker™ tubes, the High Capacity cDNA Reverse Transcription Kit and the RNase inhibitor were obtained from Invitrogen/Thermo Fisher Scientific via Life Tech Austria (Vienna, Austria). The peqGOLD total RNA kit was purchased from VWR (Vienna, Austria). Lipofectamine™ LTX with PLUS™ reagent was purchased from Fisher Scientific (Vienna, Austria). LPEI (linear polyethylenimine) transfection reagent was kindly provided by Prof. Manfred Ogris (Department of Pharmaceutical Chemistry, University of Vienna, Vienna, Austria). Fluoromount™ aqueous mounting medium, cholesterol, L-α-lysophosphatidylcholine type I (from egg yolk), oleic acid, sodium taurocholate hydrate and Dextran blue (from Leuconostoc spp.) were obtained from Sigma-Aldrich (Vienna, Austria). Precision cover slips (Ø 18 mm, borosilicate glass, 0.17 ± 0.005 mm) and microscope slides were obtained from Carl Roth (Karlsruhe, Germany). 2-Oleoylglycerol was purchased from Cayman Chemical via Sanova (Vienna, Austria). The EGFP expression plasmid (pEGFP-N1) was purchased from Clontech Laboratories (Mountain View, CA, USA). The hLXRα (human LXRα, full length; pCMV) and the hLXRβ (human LXRβ, full length; pcDNA3.1+) expression plasmids were obtained from Missouri S&T cDNA Resource Center (Rolla, MO, USA). The ABCA1_Luc (Luciferase reporter vector driven by the ABCA1 promoter; pGL4.14) was kindly provided by Dr. Ira G. Schulman (University of Virginia Health System, Charlottesville, VA, USA) [81]. The hLXRα_Gal4 (chimeric expression of hLXRα-LBD/Gal4-DBD; pCMX), hLXRβ_Gal4 (chimeric expression of hLXRβ-LBD/Gal4-DBD; pCMX) and the UAS_Luc (Gal4 responsive Luciferase reporter vector; pTK-MH100x4-Luc) plasmids were kindly provided by Prof. Makoto Makishima (Nihon University School of Medicine, Tokyo, Japan). The primary antibody against ABCA1 (polyclonal, rabbit, #NB400–105) was purchased from Novus Biologicals (Vienna, Austria). The primary antibody against Actin (monoclonal, mouse, #691001) was acquired from MP Biomedicals via Fisher Scientific Austria (Vienna, Austria). The horseradish-peroxidase conjugated anti-rabbit secondary antibody (#7074) as well as the horseradish-peroxidase conjugated anti-mouse secondary antibody (#7076) were purchased from Cell Signaling via New England Biolabs (Frankfurt am Main, Germany). The Alexa Fluor 594 conjugated anti-ZO-1 antibody (monoclonal, mouse, #339194), the primary antibodies against ABCG5 (polyclonal, rabbit, #PA5–53439), NPC1L1 (polyclonal, rabbit, #PA1–16800) and ABCB1 (polyclonal, rabbit, #PA5–82006) and the Alexa Fluor 488 conjugated goat anti-rabbit secondary antibody (polyclonal, #A-11008) were obtained from Thermo Fisher Scientific (Vienna, Austria). ABCA1 (Hs_ABCA1_1_SG QuantiTect Primer Assay), ABCG5 (Hs_ABCG5_1_SG QuantiTect Primer Assay), NPC1L1 (Hs_NPC1L1_1_SG QuantiTect Primer Assay), ABCB1 (Hs_ABCB1_1_SG QuantiTect Primer Assay) and GAPDH (Hs_GAPDH_1_SG QuantiTect Primer Assay) primers were purchased from Qiagen (Vienna, Austria). Stock solutions of the compounds were prepared in DMSO, except for digitonin, which was dissolved in ethanol. Final concentrations of DMSO did not exceed 0.1%, and final concentrations of ethanol did not exceed 1%.

#### Composition of Media, Buffers and Reagents

Complete growth medium consisted of EMEM, supplemented with 20% heat-inactivated FBS, 2 mM L-glutamine and 1x NEAA. Cryopreservation medium was composed of 70% EMEM, 20% heat-inactivated FBS and 10% DMSO. Apical medium consisted of EMEM, supplemented with 2 mM L-glutamine, 1x NEAA, 100 U ml^− 1^ penicillin and 100 μg ml^− 1^ streptomycin. Basolateral medium was composed of EMEM, supplemented with 20% heat-inactivated FBS, 2 mM L-glutamine, 1x NEAA, 100 U ml^− 1^ penicillin and 100 μg ml^− 1^ streptomycin. Serum-free DMEM was DMEM medium supplemented with 2 mM L-glutamine, 1x NEAA, 100 U ml^− 1^ penicillin and 100 μg ml^− 1^ streptomycin. DMEM with 0.5% BSA was serum-free DMEM mentioned above, additionally supplemented with 0.5% BSA. DMEM complete growth medium consisted of DMEM medium supplemented with 20% heat-inactivated FBS, 2 mM L-glutamine, 1x NEAA, 100 U ml^− 1^ penicillin and 100 μg ml^− 1^ streptomycin. DMEM with charcoal stripped FBS was serum-free DMEM mentioned above, additionally supplemented with 5% charcoal stripped FBS.

PBS (phosphate buffered saline, pH 7.4) contained 123 mM NaCl, 10.4 mM Na_2_HPO_4_, 3.16 mM KH_2_PO_4_. The NP40 lysis buffer consisted of ddH_2_O containing 150 mM NaCl, 50 mM HEPES and 1% NP40. The pH was adjusted to 7.4. The 3x sample buffer contained 187.5 mM TRIS-HCl (pH 6.8), 0.2 M SDS, 30% glycerol and 0.2 mM bromophenol blue. The 3x sample buffer containing β-mercaptoethanol consisted of 85% 3x sample buffer and 15% β-mercaptoethanol. TBST (pH 8.0) was composed of 25 mM TRIS-base, 190 mM NaCl and 0.1% Tween-20 in ddH_2_O. The ECL reagent consisted of 100 mM TRIS-base (pH 8.5), 1.24 mM luminol, 0.2 mM p-coumaric acid and 0.009% H_2_O_2_ in ddH_2_O. The trypsin/EDTA mixture contained 0.05% trypsin and 0.02% Na_2_EDTA. The crystal violet solution was composed of 1% crystal violet in 20% methanol and was filtered before use. The sodium citrate:ethanol solution consisted of 0.05 M sodium citrate in 50% ethanol. The ATP solution contained 20 mM tricine (pH 7.8), 21.5 mM MgCl_2_ and 3.8 mM adenosine-5′-triphosphate disodium salt. The luciferin solution consisted of 32 mg/ml luciferin and 21 mM tricine (pH 7.8).

##### Protocol #1: Subculturing of Caco-2 Cells

1. Pre-warm PBS, complete growth medium and trypsin/EDTA for 30 min in a 37 °C water bath.

2. Pour off the medium and discard it.

3. Rinse the cells with 12 ml PBS and discard it. Note: Volumes are given for 75 cm^2^ flasks!

4. Add 3 ml trypsin/EDTA to the flask and incubate for 5 min in an incubator at 37 °C.

5. Knock to the sides of the flask to detach still adherent cells and then add 7 ml of complete growth medium to neutralize the trypsin/EDTA solution.

6. Gently pipet up and down to separate cells and avoid cell clumps.

7. Remove 1 ml of the cell suspension for cell counting and viability measurement.

8. Transfer 1 × 10^6^ cells (Monday) or 0.5 × 10^6^ cells (Thursday) to a new 75 cm^2^ flask and fill up with complete growth medium to a total volume of 15 ml. Note: Using these cell densities enables subculturing only twice per week (Monday and Thursday). However, medium has to be renewed every other day (3 times per week).

We use Caco-2 cells between in-house passage numbers 5 to 20 only.

##### Protocol #2: Cultivation of Caco-2 Cells on Filter Inserts – Spontaneous Differentiation

Seeding of Caco-2 cells onto filter inserts is preferably done on Thursdays. Translucent inserts should be used, except for confocal laser scanning microscopy and viability assays where transparent inserts are required. Note: Volumes are given for 12-well plates with inserts from Sarstedt (growth area = 1.1 cm^2^).

1. Pre-warm PBS, complete growth medium and trypsin/EDTA for 30 min in a 37 °C water bath.

2. Pour off the medium and discard it.

3. Rinse the cells with 12 ml PBS and discard it. Note: Volumes are given for 75 cm^2^ flasks!

4. Add 3 ml trypsin/EDTA to the flask and incubate for 5 min in an incubator at 37 °C.

5. Knock to the sides of the flask to detach still adherent cells and then add 7 ml of complete growth medium to neutralize the trypsin/EDTA solution.

6. Gently pipet up and down to separate cells and avoid cell clumps.

7. Remove 1 ml of the cell suspension for cell counting and viability measurement.

8. Pre-wet the filter inserts and wells with complete growth medium for at least 2 min. Volumes used are: apical (insert) 0.5 ml and basolateral (well) 1.6 ml.

9. One insert has to be cultivated without cells, since this is required for the measurement of the TEER (transepithelial electrical resistance) blank value. Note: Consult Supplementary Protocol #3 (Additional file 1) for further information on TEER measurement.

10. Prepare the cell suspension required for seeding in complete growth medium. 0.06 × 10^6^ cells/cm^2^ should be seeded onto the filter inserts in a volume of 0.5 ml.

11. Aspirate the medium in the inserts you want to seed the cells onto.

12. Seed cells into the inserts and gently shake the plate to evenly distribute cells.

13. Put the plate into an incubator at 37 °C and 5% CO_2_.

14. Change the medium 3 h after seeding to basolateral medium, both in the insert and the well.

15. Again change the medium the next day to avoid multilayer formation. Hereafter, medium has to be renewed every other day (3 times per week) till the end of the cultivation period, which is usually after 19–21 days. Note: Caco-2 cells are grown under symmetric conditions (i.e. basolateral medium in both the filter insert and the well) for 7 days. Change to asymmetric conditions on day 7 of cultivation (i.e. apical medium in the filter insert, basolateral medium in the well). Apical medium is serum-free, whereas basolateral medium contains 20% FBS. This resembles more closely the physiological conditions.

##### Protocol #3: Luciferase Reporter Gene Assay with Caco-2 Cells

Day 1 afternoon: (e.g. Monday 15:00)

1. Pre-warm PBS, complete growth medium and trypsin/EDTA in a 37 °C water bath.

2. Pour off the medium and discard it.

3. Rinse the cells with 12 ml PBS and discard it. Note: Volumes are given for 75 cm^2^ flasks!

4. Add 3 ml trypsin/EDTA to the flask and incubate for 5 min in an incubator at 37 °C.

5. Knock to the sides of the flask to detach still adherent cells and then add 7 ml of complete growth medium to neutralize the trypsin/EDTA solution.

6. Spin cells down at 1400 rpm for 4 min at room temperature.

7. Resuspend the cell pellet in 10 ml DMEM complete growth medium by gently pipetting up and down.

8. Remove 1 ml of the cell suspension for cell counting and viability measurement.

9. Plate cells in a 96-well plate at a density of 0.02 × 10^6^ cells/well in 100 μl DMEM complete medium. Note: Cells should be approximately 40–60% confluent when transfected.

10. Incubate overnight in an incubator at 37 °C and 5% CO_2_.

Day 2 morning: (e.g. Tuesday 11:00)

1. ½ h to 1 h prior transfection aspirate old medium and add 100 μl OptiMEM reduced serum medium.

2. Prepare plasmid DNA/PLUS solution and Lipofectamine™ LTX solution separately in OptiMEM in a volume ratio of 1:1, before combining the two solutions, according to the manufacturer’s instructions.

- Total DNA: 0.1 μg/well
- PLUS™ reagent: 0.1 μl/well
- Lipofectamine™ LTX: 0.5 μl/well.

First dilute the plasmid DNA in OptiMEM, then add the PLUS™ reagent. Then dilute Lipofectamine™ LTX in OptiMEM and then transfer the plasmid DNA/PLUS solution to the Lipofectamine™ LTX solution.

3. Mix by pipetting up and down several times.

4. Incubate for 5 min at room temperature.

5. Add 10 μl of the DNA/Lipofectamine mixture to each well and mix by shaking the plate carefully.

6. Incubate for 24 h in an incubator at 37 °C and 5% CO_2_.

Day 3 morning: (e.g. Wednesday 11:00)

1. Prepare compound dilutions in DMEM with 5% charcoal stripped FBS in 2-fold concentration shortly prior to use.

2. Aspirate old medium from the plate and add 50 μl of DMEM with charcoal stripped FBS to each well. Then add 50 μl of the prepared compound dilutions. The final volume is 100 μl/well and onefold compound concentration.

3. Incubate for 24 h in an incubator at 37 °C and 5% CO_2_.

Day 4 morning: (e.g. Thursday 11:00)

1. Prepare Luciferase lysis buffer: 4.8 ml H_2_Odd, 1.2 ml 5x lysis buffer, 6 μl CoA (coenzyme A trilithium salt, 270 mM), 6 μl DTT (dithiothreitol, 1 M)

2. Aspirate old medium and add 50 μl luciferase lysis buffer/well. Freeze at − 80 °C to enhance cell lysis for at least 1 h and max. up to 1 week.

3. For measurement, remove the plate from the freezer and let it thaw (app. ½ h).

4. Shake it for 5 min. Then transfer 40 μl lysed cell solution to a black 96-well plate. Shake the plate briefly (1 min.) to prevent bubbles within the wells.

5. The plate is now ready for measurement. Measure fluorescence first, at an excitation wavelength of 485 nm and an emission wavelength of 520 nm, if EGFP is used as internal control. After the injection of ATP (50 μl) and luciferin (50 μl) solutions measure luminescence. Normalize the luciferase-derived luminescence to the EGFP-derived fluorescence in order to account for differences in cell numbers.

##### Protocol #4: Cholesterol Uptake Assay

Cells should be cultivated and fully differentiated on translucent filter inserts as described in Protocol #2. Before starting the experiments, measure TEER in order to assure the integrity of the cell monolayer. Note: Volumes are given for 12-well plates with inserts from Sarstedt (growth area = 1.1 cm^2^).

Day 1 before lunchtime (e.g. Monday 11:30):

1. Pre-warm PBS, serum-free DMEM and DMEM with 0.5% BSA in a 37 °C water bath.

2. Carefully aspirate medium from both the wells and the filter inserts.

3. Wash both the well and the filter insert at least once with PBS.

4. Put 0.5 ml of serum-free DMEM into each filter insert. Put 1.6 ml of DMEM with 0.5% BSA into each well.

5. Incubate for 24 h in an incubator at 37 °C and 5% CO_2_.

Day 2 before lunchtime (e.g. Tuesday 11:30):

1. Pre-warm PBS and serum-free DMEM in a 37 °C water bath.

2. Prepare compound dilutions in serum-free DMEM in 2-fold concentration shortly prior to use.

3. Carefully aspirate medium from both the wells and the filter inserts.

4. Wash at least once with PBS.

5. Put 0.25 ml serum-free DMEM into the filter inserts. Fill the wells with 1.6 ml of serum-free DMEM.

6. Then add 0.25 ml of the respective compound dilutions into the filter inserts.

7. Incubate for 48 h in an incubator at 37 °C and 5% CO_2_.

Day 4 morning (e.g. Thursday 9:00):

1. Start with the Preparation of Stock Solutions of Substances for Micelles

Note: Micelles simulating postprandial conditions contain cholesterol, sodium taurocholate, lysophosphatidylcholine, oleic acid and 2-oleoylglycerol. Final concentrations of these substances should be as follows: cholesterol 0.05 mM, sodium taurocholate 2 mM, lysophosphatidylcholine 0.2 mM, oleic acid 0.6 mM and 2-oleoylglycerol 0.2 mM [35, 36].

Note: As treatment and application of micelles is required in parallel, the substances mentioned above are prepared in 2-fold concentration and then diluted by the treatment to onefold. To prepare these 2-fold concentrations, stock solutions are prepared first [35, 36], with the concentrations stated in Table 1.

**Table 1:** Components for micelles and concentrations [35, 36] of the stock solutions

Substance	2-fold concentration	Concentration of stock solution
Cholesterol	0.1 mM	25 mM
Sodium taurocholate	4 mM	24 mM
Lysophosphatidylcholine	0.4 mM	100 mM
Oleic acid	1.2 mM	100 mM
2-Oleoylglycerol	0.4 mM	100 mM

Prepare stock solutions in chloroform:methanol 2:1 (v/v), except for the sodium taurocholate stock solution, which is prepared in serum-free DMEM.

2. Pipet the required amount of each stock solution, except for the sodium taurocholate stock solution, into a sterile glass vial with a screw-cap.

3. Then add the radioactively labelled cholesterol. The required final onefold activity is 1 μCi/ml (37 kBq/ml).

4. Evaporate this mixture to dryness under a gentle stream of nitrogen.

5. Add the required amount of sodium taurocholate stock solution and fill up with serum-free DMEM to achieve the desired final volume for 2-fold concentration.

6. Vortex the mixture vigorously for 5 min and then place it under a heating hood set at 37 °C on an orbital shaker (set at 100 rpm) for 1 h.

7. In the meantime, prepare treatment and serum-free DMEM containing 1% human plasma (serves as acceptor on the basolateral side).

8. Filter sterilize the micellar solution through a 0.45 μm cellulose acetate filter.

9. Aspirate old medium from the plate. Put 1.6 ml of DMEM with human plasma into the wells. Put 0.25 ml micellar solution into the filter inserts.

10. Then pipet 0.25 ml of compound treatment into the filter inserts. Note: Treatment shall be carried out for 48 h and then micelles and fresh treatment shall be added for another 2 h. Make sure to be on time.

11. Place the plate on an orbital shaker (set at 200 rpm) in an incubator at 37 °C and 5% CO_2_ for 2 h.

12. Hereafter, carefully aspirate the basolateral and apical medium. Then wash at least two times with PBS.

13. Lyse the cells with 150 μl NP40 lysis buffer/insert for 30 min at room temperature.

14. Scratch the cells to the edge of the filter insert with the help of an inoculation needle. Be careful not to rip the filter membrane.

15. Transfer the cell lysates into centrifuge tubes. Centrifuge the tubes at 13000 rpm at 4 °C for 20 min.

16. After centrifuging, transfer the supernatants into new centrifuge tubes.

17. Fill scintillation vials with 3.5 ml scintillation liquid and appropriately mark them.

18. Pipet 50 μl of each cell lysate into a separate scintillation vial. As a control, pipet 250 μl of micellar solution that was applied to the inserts into a scintillation vial.

19. Then measure dpm (disintegrations per minute) with the help of a liquid scintillation counter.

Day 5 (e. g.Friday):

1. Perform a Bradford assay or equivalent in order to determine protein concentration in the cell lysates. This is required for expressing the results as dpm/mg protein or nmol cholesterol/mg protein.

##### Protocol #5: Cholesterol Efflux Assay

Cells should be cultivated and fully differentiated on translucent filter inserts as described in Protocol #2. Before starting the experiments, measure TEER in order to assure the integrity of the cell monolayer. Note: Volumes are given for 12-well plates with inserts from Sarstedt (growth area = 1.1 cm^2^).

Day 1 morning (e.g. Sunday 10:00):

1. Pre-warm PBS, serum-free DMEM and DMEM with 0.5% BSA in a 37 °C water bath.

2. Carefully aspirate medium from both the wells and the filter inserts.

3. Wash both the well and the filter insert at least once with PBS.

4. Put 0.5 ml of serum-free DMEM into each filter insert. Put 1.6 ml of DMEM with 0.5% BSA into each well.

5. Incubate for 24 h in an incubator at 37 °C and 5% CO_2_.

Day 2 morning (e.g. Monday 08:30):

1. Prepare stock solutions and micelles, as detailed in Protocol #4.

2. Filter sterilize the micellar solution through a 0.45 μm cellulose acetate filter.

3. Aspirate old medium from the plate. Wash cells once with PBS. Put 1.6 ml of serum-free DMEM into the wells and 0.25 ml of serum-free DMEM into the inserts.

4. Then pipet 0.25 ml of the micellar solution into the filter inserts.

5. Place the plate on an orbital shaker (200 rpm) in an incubator at 37 °C and 5% CO_2_ for 24 h.

Day 3 morning (e.g. Tuesday 10:00):

1. Pre-warm PBS and serum-free DMEM in a 37 °C water bath.

2. Prepare compound dilutions in serum-free DMEM in 2-fold concentration shortly prior to use.

3. Carefully aspirate medium from both the wells and the filter inserts.

4. Wash cells once with PBS.

5. Put 0.25 ml serum-free DMEM into the filter inserts. Fill the wells with 1.6 ml of serum-free DMEM.

6. Then add 0.25 ml of the respective compound dilutions into the filter inserts.

7. Incubate for 48 h on an orbital shaker (200 rpm) in an incubator at 37 °C and 5% CO_2_.

Day 5 morning (e.g. Thursday 08:30):

1. Pre-warm serum-free DMEM in a 37 °C water bath.

2. Prepare serum-free DMEM containing 1% human plasma (serves as acceptor on the basolateral side).

3. Prepare micelles as described for day 2, but without adding any cholesterol.

3. Carefully aspirate medium from both the wells and the filter inserts.

4. Put 0.25 ml serum-free DMEM into the filter inserts. Fill the wells with 1.6 ml of serum-free DMEM containing human plasma. Then pipet 0.25 ml of the micellar solution into the filter inserts.

5. Incubate for 24 h on an orbital shaker (set at 200 rpm) in an incubator at 37 °C and 5% CO_2_.

Day 6 morning (e.g. Friday 10:00):

1. Carefully collect the apical and basolateral supernatants by pipetting them off and put them into 2.0 ml centrifuge tubes.

2. Centrifuge for 5 min at 13000 rpm at 4 °C. Transfer the supernatants into new centrifuge tubes.

3. Wash the cells two times with PBS.

4. If you want to show the amount of cholesterol remaining in the cells, lyse the cells with 150 μl NP40 lysis buffer/insert for 30 min at room temperature.

5. Scratch the cells to the edge of the filter insert with the help of an inoculation needle. Be careful not to rip the filter membrane.

6. Transfer the cell lysates into centrifuge tubes. Centrifuge the tubes at 13000 rpm at 4 °C for 20 min.

7. After centrifuging, transfer the supernatants into new centrifuge tubes.

8. Fill scintillation vials with 3.5 ml scintillation liquid and appropriately mark them.

9. Pipet 50 μl of each cell lysate, 250 μl of each apical supernatant and 800 μl of each basolateral supernatant into a separate scintillation vial.

10. Then measure dpm (disintegrations per minute) with the help of a liquid scintillation counter.

11. Perform a Bradford assay or equivalent in order to determine protein concentration in the cell lysates. Normalize the counts in the cell lysates to the protein content, if you want to show the amount of cholesterol remaining in the cells. In the final analysis, consider that the volumes of the apical supernatant, the basolateral supernatant and the cell lysate are unequal and include a factor accounting for this disparity.

##### Protocol #6: Determination of Protein Level of Major Cholesterol Transporters - Confocal Microscopy for ABCG5, NPC1L1 and ABCB1

Cells should be cultivated and fully differentiated on transparent filter inserts as described in Protocol #2. Before starting the experiments, measure TEER in order to assure the integrity of the cell monolayer. Note: Volumes are given for 12-well plates with inserts from Sarstedt (growth area = 1.1 cm^2^).

Day 1 and Day 2 of this protocol are identical to Supplementary Protocol #5 (Additional file 1).

Day 4 morning (e.g. Thursday 09:00):

1. Carefully aspirate medium from both the wells and the filter inserts.

2. Wash cells two times with PBS.

3. Fix cells with 3.7% formaldehyde in PBS for 15 min at room temperature.

4. Aspirate formalin and wash cells two times with PBS.

5. Permeabilize cells with 0.2% Triton X in PBS for 10 min at room temperature.

6. Wash cells two times with PBS.

7. Add blocking solution (5% BSA in PBS) to both the insert and the well for 6 h. Put the plate at 4 °C.

8. Prepare a dilution of the primary antibody in PBS with 1% BSA according to the manufacturer’s instructions (ABCG5: 2 μg/ml (1150), NPC1L1: 10 μg/ml (1:100), ABCB1: 2 μg/ml (1100)).

9. Wash two times with PBS. Add 500 μl of the primary antibody working solution to the inserts and incubate overnight at 4 °C.

Day 5 morning (e.g. Friday 09:00):

1. Prepare a dilution of the secondary antibody in PBS with 1% BSA according to the manufacturer’s instructions (Alexa Fluor 488 conjugated goat anti-rabbit antibody: 4 μg/ml (1400)).

2. Wash two times with PBS. Add 500 μl of the secondary antibody working solution to the inserts and incubate for 3 h at 4 °C in the dark.

3. Prepare a dilution of DAPI in PBS with 0.2% BSA (1 μg/ml (11000)).

4. Wash two times with PBS. Add 500 μl of the DAPI working solution to the inserts and incubate for 15 min at room temperature in the dark.

5. Wash two times with PBS.

6. Put one drop of mounting medium on a microscope slide.

7. Then carefully cut the filter membrane from the plastic insert with the help of a scalpel. Carefully place the membrane upside down in the mounting medium, avoiding air bubbles.

8. Put one drop of mounting medium onto the membrane and then cover it with a cover slip, again avoiding air bubbles.

9. Put the microscope slides into a box and store the box at 4 °C in the dark while the mounting medium is drying off.

Samples are now ready for inspection with the confocal laser scanning microscope.

##### Protocol #7: Viability Assays - Resazurin Conversion Assay and Subsequent Crystal Violet Staining

Cells should be cultivated and fully differentiated on transparent filter inserts as described in Protocol #2. Before starting the experiments, measure TEER in order to assure the integrity of the cell monolayer. Note: Volumes are given for 12-well plates with inserts from Sarstedt (growth area = 1.1 cm^2^).

Day 1 and Day 2 of this protocol are identical to Supplementary Protocol #5 (Additional file 1).

Day 4 morning (e.g. Thursday 09:00):

1. Pre-warm serum-free DMEM in a 37 °C water bath.

2. Prepare a dilution of resazurin in serum-free DMEM at 10 μg/ml concentration.

3. Carefully aspirate medium from both the wells and the filter inserts.

4. Wash at least once with serum-free DMEM.

5. Add 0.5 ml of the resazurin dilution to the filter inserts and 1.6 ml to the wells.

6. Incubate for 2 h in an incubator at 37 °C and 5% CO_2_.

7. Using forceps, carefully transfer the filter inserts into a new 12-well plate.

8. Remove 400 μl of apical medium from each filter insert and pipet it into the wells of a 48-well plate.

9. Measure fluorescence on a plate reader, using an excitation of 535 nm and an emission of 590 nm.

10. Carefully aspirate remaining resazurin dilution from the filter inserts.

11. Add 500 μl of crystal violet solution per insert and incubate for 10 min at room temperature.

12. Using forceps, pull the insert out of the well one by one and carefully wash it with deionized water. Repeat this washing step till the water is colorless (3–5 times).

13. Carefully aspirate remaining water from both the inserts and the wells and let the inserts dry overnight at room temperature.

Day 5 morning (e.g. Friday 09:00):

1. Add 500 μl sodium citrate:ethanol per insert.

2. Carefully shake for 10 s.

3. Add another 500 μl sodium citrate:ethanol per insert.

4. Again shake for 10 s.

5. Pipet 100 μl out of each insert into a 96-well plate and measure OD at 595 nm.

### Statistical Analysis

GraphPad Prism™ 6.01 was used for statistical analysis and the generation of figures. Differences between two groups were analyzed by a two-tailed paired t-test. Multiple comparisons were carried out by one-way ANOVA with Dunnett post-test. *P* < 0.05 was considered as statistically significant.

## Additional Files

**Additional file 1: Supplementary Table 1.** Equipment used and respective manufacturer. **Supplementary Table 2.** Software used for data acquisition and analysis. **Supplementary Protocol #1.** Thawing and maintenance of Caco-2 cells. **Supplementary Protocol #2.** Freezing of Caco-2 cells. **Supplementary Protocol #3.** Quality control steps during differentiation and experiments with Caco-2 cells - TEER measurement. **Supplementary Protocol #4.** Paracellular permeability of Dextran Blue and [^14^C]-mannitol. **Supplementary Protocol #5.** Determination of protein level of major cholesterol transporters - Western Blotting for ABCA1. **Supplementary Protocol #6.** Determination of mRNA level of major cholesterol transporters - qRT-PCR for ABCA1, ABCG5, NPC1L1 and ABCB1.

## Data Availability

The datasets used and/or analyzed during the current study are available from the corresponding author on reasonable request.
