# Supplementary material for: Caco-2 Cells for Measuring Intestinal Cholesterol Transport - Possibilities and Limitations
Source: Biol Proced Online. 2020 Apr 11;22:7. doi: 10.1186/s12575-020-00120-w (PMC7149936; doi:10.1186/s12575-020-00120-w)
Supplement: Supplementary file 1 — Additional file 1: Supplementary Table 1. Equipment used and respective manufacturer. Supplementary Table 2. Software used for data acquisition and analysis. Supplementary Protocol #1. Thawing and maintenance of Caco-2 cells. Supplementary Protocol #2. Freezing of Caco-2 cells. Supplementary Protocol #3. Quality control steps during differentiation and experiments with Caco-2 cells - TEER measurement. Supplementary Protocol #4. Paracellular permeability of Dextran Blue and [14C]-mannitol. Supplementary Protocol #5. Determination of protein level of major cholesterol transporters - Western Blotting for ABCA1. Supplementary Protocol #6. Determination of mRNA level of major cholesterol transporters - qRT-PCR for ABCA1, ABCG5, NPC1L1 and ABCB1. [file 12575_2020_120_MOESM1_ESM.docx]

**Supplementary information:**

**Caco-2 cells for measuring intestinal cholesterol transport - possibilities and limitations**

Verena Hiebl^1^, Daniel Schachner^1^, Angela Ladurner^1^, Elke H. Heiss^1^, Herbert Stangl^2^, Verena M. Dirsch^1,^ *

^1^Department of Pharmacognosy, University of Vienna, Althanstrasse 14, 1090 Vienna, Austria.

^2^Institute of Medical Chemistry, Center for Pathobiochemistry and Genetics, Medical University of Vienna, Vienna, Austria

*Corresponding author: Prof. Dr. Verena M. Dirsch, email: verena.dirsch@univie.ac.at

T: 0043 1 4277-55270; University of Vienna, Department of Pharmacognosy, Althanstrasse 14, 1090 Vienna, Austria;

**Supplementary Table 1: Equipment used and respective manufacturer.**

| **Equipment** | **Manufacturer** |
| --- | --- |
| Heraeus™ Multifuge 1 S-R | Kendro Laboratory Products (Langenselbold, Germany) |
| ViCell™ XR cell viability analyzer | Beckman Coulter (Brea, CA, USA) |
| Heracell™ 150 Incubator (37 °C and 5% CO_2_) | Thermo Electron Corporation (Waltham, MA, USA) |
| EVOM2 epithelial voltohmmeter | World Precision Instruments Germany (Berlin, Germany) |
| Tecan Sunrise™ plate reader | Tecan (Mannedorf, Switzerland) |
| Tri-carb® 2910TR liquid scintillation counter | Perkin Elmer (Waltham, MA, USA) |
| Tecan Spark™ plate reader | Tecan (Mannedorf, Switzerland) |
| Heraeus™ Biofuge fresco | Kendro Laboratory Products (Langenselbold, Germany) |
| LAS-3000™ Luminescent Image Analyzer | Fujifilm (Tokyo, Japan) |
| Leica DMi8 Confocal Laser Scanning Microscope | Leica Microsystems (Wetzlar, Germany) |
| Eppendorf® Thermomixer compact | Eppendorf AG (Hamburg, Germany) |
| NanoDrop 2000c | Thermo Fisher Scientific (Waltham, MA, USA) |
| C1000™ Thermal Cycler | BIO-RAD Laboratories (Hercules, CA, USA) |
| LightCycler® 480 | Roche (Basel, Switzerland) |
| Swip SM25 DIGI orbital shaker | Edmund Buehler GmbH (Bodelshausen, Germany) |
| Thermo Scientific™ CO_2_ resistant orbital shaker (#88881102) | Thermo Fisher Scientific (Waltham, MA, USA) |

**Supplementary Table 2: Software used for data acquisition and analysis.**

| **Software** |
| --- |
| ViCell™ XR 2.03 |
| Tecan XFLUOR4 V4.51 |
| QuantaSmart™ V4.00 |
| Tecan SparkControl™ V2.1 |
| Image Reader LAS-3000™ V2.1 |
| MultiGauge V3.0 Science Lab 2005 |
| Leica LASX |
| NanoDrop 2000 software V1.4.2 |
| LightCycler® 480 software V1.5.1.62 |
| GraphPad Prism™ 6.01 |

**Supplementary Protocol #1: Thawing and maintenance of Caco-2 cells**

1. Add 8.5 ml of pre-warmed complete growth medium to a conical centrifuge tube.

2. Thaw a vial of frozen Caco-2 cells in a water bath at 37 °C. Rapidly proceed when most of the content has melted.

3. Transfer the cells to the conical centrifuge tube, slowly and carefully, using aseptic techniques.

4. Centrifuge at 1400 rpm at room temperature for 8 minutes.

5. Carefully aspirate or pour off the medium, without disturbing the pellet.

6. Add 10 ml of complete growth medium and resuspend the pellet by gently pipetting up and down.

7. Remove 1 ml of the cell suspension for cell counting and viability measurement.

8. Add 1 × 10^6^ cells to a 75 cm^2^ flask and fill up with complete growth medium to a total volume of 15 ml.

9. Transfer the flask to an incubator at 37 °C and 5% CO_2_.

10 Inspect the culture daily by eye and under a microscope to ensure that the culture is free of contamination.

11. Change medium 24 h after thawing and hereafter every other day (3 times per week), or subculture cells if needed. **Note:** Subculturing of cells is required at 80% confluence!

**Supplementary Protocol #2: Freezing of Caco-2 cells**

1. Perform subculturing of the cells as described in Protocol #1.

2. After removing 1 ml of the cell suspension for cell counting and viability measurement, centrifuge remaining cells at 1400 rpm at room temperature for 8 minutes.

3. Calculate the amount of cryopreservation medium needed to achieve a concentration of 2 × 10^6^ cells per 1.5 ml. Prepare cryopreservation medium and put it on ice.

4. Resuspend the cell pellet in the calculated amount of cryopreservation medium by gently pipetting up and down, then put on ice.

5. Dispense 1.5 ml of the prepared cell suspension into an appropriately marked 2 ml cryovial and put it on ice. Continue like that with the rest of the cell suspension. **Note:** A minimum equilibration time of 10 minutes is required for the DMSO to penetrate the cells! However, longer exposure to DMSO is toxic to the cells and might affect cell viability!

6. Transfer the cryovials into a styrofoam box and put it into a -80 °C freezer.

7. After 2 – 3 days, the cryovials have to be transferred into the liquid nitrogen tank for long term storage. **Note:** This transfer has to be done quickly in order to avoid warming, as this could affect the viability of the cell culture!

**Supplementary Protocol #3: Quality control steps during differentiation and experiments with Caco-2 cells - TEER measurement**

Volumes are given for 12-well plates with inserts from Sarstedt (growth area = 1.1 cm^2^).

Before making any measurements, charge the TEER voltohmmeter overnight. When you start the disinfection, make sure that the voltohmmeter is disconnected from the charger. The protocol below follows in basic the manufacturer’s instructions and includes volume adjustments for the inserts and plates used.

1. Pre-warm apical medium and sterile ddH_2_O in a 37 °C water bath.

2. In the meantime, bring the TEER voltohmmeter and the TEER electrode into the laminar airflow. **Note:** Do not spray neither the voltohmmeter nor the electrode with ethanol!!

3. Prepare a conical centrifuge tube with app. 25 ml 70% ethanol and place the unplugged electrode in this tube. Wait for 20 minutes. While disinfecting the electrode, switch on the voltohmmeter to allow it to warm up.

4. Prepare two conical centrifuge tubes with app. 20 ml of sterile ddH_2_O. Prepare one centrifuge tube with app. 20 ml of apical medium.

5. Pull the electrode out of the tube with ethanol and let it air dry for a few seconds.

6. Next place the electrode in the first centrifuge tube with ddH_2_O and gently swirl it to wash remaining ethanol off.

7. Then place the electrode in the second centrifuge tube with ddH_2_O and again gently swirl it.

8. Next place the electrode in the centrifuge tube containing the apical medium.

9. Set the function switch on the voltohmmeter to Ohms. Insert the plug at the end of the electrode cable into the input port on the voltohmmeter.

10. Wait for app. 10 minutes. In the meantime, pipet 300 µl of apical medium into the inserts of your plate and put it back into the incubator. **Note:** Crucial step! It has to be ensured that the shorter electrode is fully inserted into the medium, otherwise the measurement is not accurate.

11. Now you are ready for resistance measurements. Take the plate out of the incubator. Start with measuring the blank resistance (insert without cells) and continue measuring the resistance of the inserts with cells. The shorter electrode always has to be placed in the insert, while the longer electrode is placed in the well and touches the bottom of the well.

12. When you have finished your measurements, unplug the electrode from the voltohmmeter and switch the voltohmmeter off.

13. Rinse the electrode first with ddH_2_O, second with 70% ethanol and finally again with ddH_2_O.

14. Store the electrode dry in a dark place.

To assure proper measurements, TEER electrodes have to be cleaned routinely. Furthermore, instrument diagnostics should be performed on a routine basis. To do so, consult the instruction manual of the manufacturer.

We only use inserts with a TEER value of ≥ 200 Ω cm^2^ at 37 °C for experiments.

**Supplementary Protocol #4: Paracellular permeability of Dextran Blue and [^14^C]-mannitol**

Cells should be cultivated and fully differentiated on translucent filter inserts as described in Protocol #2. Before starting the experiments, measure TEER in order to assure the integrity of the cell monolayer. **Note:** Volumes are given for 12-well plates with inserts from Sarstedt (growth area = 1.1 cm^2^).

Day 1 morning (e.g. Monday 09:00):

1. Pre-warm PBS, serum-free DMEM and DMEM with 0.5% BSA in a 37 °C water bath.

2. Carefully aspirate medium from both the wells and the filter inserts.

3. Wash both the well and the filter insert at least once with PBS.

4. Put 0.5 ml of serum-free DMEM into each filter insert. Put 1.6 ml of DMEM with 0.5 % BSA into each well.

5. Incubate for 24 h in an incubator at 37 °C and 5% CO_2_.

Day 2 morning (e.g. Tuesday 09:00):

1. Pre-warm PBS and serum-free DMEM in a 37 °C water bath.

2. Carefully aspirate medium from both the wells and the filter inserts.

3. Wash at least once with PBS.

4. Put 0.5 ml serum-free DMEM into the filter inserts. Fill the wells with 1.6 ml of serum-free DMEM.

5. Incubate for 48 h in an incubator at 37 °C and 5% CO_2_.

Day 4 morning: (e.g. Thursday 09:00)

1. Prepare a Dextran Blue stock solution and a [^14^C]-mannitol stock solution. The Dextran Blue stock solution (20 mg/ml) is prepared in serum-free DMEM (without supplements) and kept at 70 °C and 1400 rpm till it dissolves (app. 60 minutes). The missing supplements are added afterwards. The [^14^C]-mannitol stock solution is prepared in serum-free DMEM yielding an activity of 0.5 µCi/ml (18.5 kBq/ml).

2. Carefully aspirate medium from both the wells and the filter inserts.

3. Put 0.25 ml serum-free DMEM into the filter inserts, then add either 0.25 ml of Dextran Blue stock solution or 0.25 ml of [^14^C]-mannitol stock solution.

4. Fill the wells with either 1.6 ml of serum-free DMEM containing 2.5% human plasma (for Dextran Blue) or with 1.6 ml of serum-free DMEM (for [^14^C]-mannitol).

5. Incubate on a shaker (200 rpm) in an incubator set at 37 °C and 5% CO_2_.

6. For Dextran Blue, a 100 µl sample from the basolateral compartment is taken every hour, until the total incubation period of 6 hours is over. Each time a sample is taken, 100 µl of serum-free DMEM containing 2.5% human plasma is added to the basolateral compartment to keep a total volume of 1.6 ml.

7. For [^14^C]-mannitol, a 100 µl sample from the basolateral compartment is taken every 20 minutes, until the total incubation period of 2 hours is over. Each time a sample is taken, 100 µl of serum-free DMEM is added to the basolateral compartment to keep a total volume of 1.6 ml.

8. For Dextran Blue, pipet the different samples into a 96-well plate and measure the absorbance at 620 nm. Always include a calibration curve on the same plate to calculate the amount of Dextran Blue that has passed through the monolayer into the basolateral compartment.

9. For [^14^C]-mannitol, measure each sample in a liquid scintillation counter (dpm). Also measure the counts in 100 µl of the stock solution in order to know the dpm applied to the apical side of the cells.

Concerning the Dextran Blue permeability, calculate the amount of Dextran Blue in percent that was found in the basolateral compartment at each time point.

The apparent permeability (P_app_) of [^14^C]-mannitol was calculated according to the following equation:

P_app_ = (steady state flux)(1/(A C_donor_)) = (dpm/min)(1/(cm^2^ dpm/ml))

In this equation, the steady state flux (in dpm/min) is normalized to the surface area of the filter insert (A, in cm^2^). C_donor_ is the initial concentration of [^14^C]-mannitol in the apical compartment (in dpm/ml).

**Supplementary Protocol #5: Determination of protein level of major cholesterol transporters - Western Blotting for ABCA1**

Cells should be cultivated and fully differentiated on translucent filter inserts as described in Protocol #2. Before starting the experiments, measure TEER in order to assure the integrity of the cell monolayer. **Note:** Volumes are given for 12-well plates with inserts from Sarstedt (growth area = 1.1 cm^2^).

Day 1 morning (e.g. Monday 09:00):

1. Pre-warm PBS, serum-free DMEM and DMEM with 0.5% BSA in a 37 °C water bath.

2. Carefully aspirate medium from both the wells and the filter inserts.

3. Wash both the well and the filter insert at least once with PBS.

4. Put 0.5 ml of serum-free DMEM into each filter insert. Put 1.6 ml of DMEM with 0.5 % BSA into each well.

5. Incubate for 24 h in an incubator at 37 °C and 5% CO_2_.

Day 2 morning (e.g. Tuesday 09:00):

1. Pre-warm PBS and serum-free DMEM in a 37 °C water bath.

2. Prepare compound dilutions in serum-free DMEM in 2-fold concentration shortly prior to use.

3. Carefully aspirate medium from both the wells and the filter inserts.

4. Wash at least once with PBS.

5. Put 0.25 ml serum-free DMEM into the filter inserts. Fill the wells with 1.6 ml of serum-free DMEM.

6. Then add 0.25 ml of the respective compound dilutions into the filter inserts.

7. Incubate for 48 h in an incubator at 37 °C and 5% CO_2_.

Day 4 morning (e.g. Thursday 09:00):

1. Prepare ready-to-use lysis buffer containing protease and phosphatase inhibitors:

NP40 lysis buffer

40:1000 cOmplete (25x stock)

10:1000 PMSF (100 mM in isopropanol)

5:1000 NaF (200 mM)

5:1000 Na_3_VO_4_ (200 mM)

Put the lysis buffer on ice.

2. Carefully aspirate the medium from both the inserts and the wells.

3. Place the plate on ice and perform further steps on ice.

4. Wash cells once with cold PBS.

5. Pipet 150 µl cold lysis buffer into each filter insert. Put the ice box with the plate on a shaker in a cold room at 4 °C for 30 minutes.

6. Using an inoculation needle, carefully scratch the cells to the edge of the filter insert, without ripping the filter membrane.

7. Carefully pipet up and down 2 – 3 times to detach still remaining cells and finally transfer the cell suspension into 1.5 ml centrifuge tubes. **Note:** The procedure can be stopped at this point by freezing the cell lysates at -80 °C.

8. Centrifuge the tubes at 13000 rpm and 4 °C for 20 minutes in order to pellet the cell debris.

9. Hereafter, carefully transfer the supernatants into new 1.5 ml centrifuge tubes, without touching the cell pellet. **Note:** The procedure can be stopped at this point by freezing the protein samples at -20 °C.

10. Perform a Bradford assay or an equivalent assay to determine the protein concentration in your samples.

11. Dilute each protein sample with 3x sample buffer containing β-mercaptoethanol (2+1, v/v) and mix by vortexing. Do not heat the samples!! **Note:** The procedure can be stopped at this point by freezing the protein samples at -80 °C.

The samples are now ready for gel electrophoresis and western blotting.

For separating proteins, a 7.5% polyacrylamide gel is used, 20 µg protein is loaded and the SDS-PAGE is carried out at constant ampere (25 mA/gel). Let the bands run out of the gel for app. 5 minutes, since ABCA1 is a ~220 kDa protein. A wet transfer is then performed with a PVDF membrane (pore size 0.2 µm). Transfer is carried out at constant volt (100 V) for 110 minutes.

The membrane is blocked for 1.5 h in TBST with 5% nonfat dry milk, washed 3x in TBST and then incubated overnight with the primary antibody (in TBST, 2 µg/ml (1:500)) at 4 °C. Incubation with the horseradish-peroxidase conjugated secondary antibody (in TBST with 5% nonfat dry milk, 1:1000) is performed for 1.5 h at room temperature.

After visualization with the help of an ECL reagent and a LAS3000 Luminescent Image Analyzer, the membrane is stripped with 0.5 N NaOH for 15 minutes. After the washing steps (3x in TBST), the membrane is incubated with the primary antibody against actin (in TBST, 1:10000) and the respective secondary antibody (in TBST with 5% nonfat dry milk, 1:1000) like done for ABCA1.

**Supplementary Protocol #6: Determination of mRNA level of major cholesterol transporters - qRT-PCR for ABCA1, ABCG5, NPC1L1 and ABCB1**

Cells should be cultivated and fully differentiated on translucent filter inserts as described in Protocol #2. Before starting the experiments, measure TEER in order to assure the integrity of the cell monolayer. **Note:** Volumes are given for 12-well plates with inserts from Sarstedt (growth area = 1.1 cm^2^).

Day 1 of this protocol is identical to Supplementary Protocol #5.

Day 2 morning (e.g. Tuesday 09:00):

1. Pre-warm PBS and serum-free DMEM in a 37 °C water bath.

2. Prepare compound dilutions in serum-free DMEM in 2-fold concentration shortly prior to use.

3. Carefully aspirate medium from both the wells and the filter inserts.

4. Wash at least once with PBS.

5. Put 0.25 ml serum-free DMEM into the filter inserts. Fill the wells with 1.6 ml of serum-free DMEM.

6. Then add 0.25 ml of the respective compound dilutions into the filter inserts.

7. Incubate for 24 h (ABCA1, ABCG5, ABCB1), 6 h (NPC1L1) or different time points in an incubator at 37 °C and 5% CO_2_.

Day 3 morning (e.g. Wednesday 09:00):

1. Carefully aspirate the medium from both the wells and the filter inserts.

2. Wash cells once with cold PBS.

2. Add 400 µl TRIzol reagent to the inserts and incubate for 10 minutes.

3. Transfer the lysates to fresh tubes and submit them 3x to a freeze/thaw cycle at -80 °C. **Note:** If you don’t want to extract the RNA right away, you can store the samples at -80 °C after this step.

4. Transfer the samples to the phasemaker™ tubes and add 80 µl chloroform per tube.

5. Shake vigorously for 15 sec. by hand.

6. Incubate for 10 – 15 minutes at room temperature.

7. Centrifuge samples for 10 minutes at 4 °C and 13000 rpm.

8. Transfer the aqueous phase (colorless, upper phase) to a fresh tube.

9. Add 10 µg RNase-free glycogen as a carrier to the aqueous phase.

10. Add 200 µl isopropanol to the aqueous phase, vortex and incubate for 10 minutes at room temperature.

11. Centrifuge for 30 minutes at 4 °C and 13000 rpm.

12. Discard the supernatant with a pipet tip.

13. Resuspend the pellet in 400 µl 75% ethanol, vortex briefly and then centrifuge at 4 °C for 5 – 10 minutes at 13000 rpm.

14. Repeat this washing step once.

15. Carefully remove the supernatant with a pipet tip.

16. Let the pellet dry for 10 minutes at 37 °C.

17. Resuspend the pellet in 20 µl RNase-free water.

18. Incubate on a 56 °C heat block for 12 minutes, to facilitate dissolving of the RNA.

19. Assess RNA concentration, as well as the quality of the extracted RNA on a Nanodrop by calculating the A260/280 (absorbance at 260 nm and 280 nm wavelength) ratio. A ratio of ~2 is considered as pure.

19. Store at -80 °C.

The samples are now ready for cDNA synthesis and real time PCR.

The cDNA synthesis is carried out with the High Capacity cDNA Reverse Transcription Kit including an RNase inhibitor, ensuing from 0.7 µg RNA. The real time PCR is performed with the SYBR Green I Master Mix with 28 ng cDNA, on a LightCycler® 480.

Results are expressed as the ratio of expression of the respective gene to that of GAPDH or another control gene.
